# Towards a comprehensive understanding of the processes underlying phenomenological continuity

**DOI:** 10.1098/rstb.2025.0162

**Published:** 2026-11-10

**Authors:** Odysse M. Davis, Lynette J. Tippett, Donna Rose Addis

**Affiliations:** ^1^ Department of Psychology, The University of Auckland, Auckland, New Zealand; ^2^ Centre for Brain Research, The University of Auckland, Auckland, New Zealand; ^3^ Rotman Research Institute, Baycrest Academy for Research and Education, Toronto, Canada; ^4^ Department of Psychology, University of Toronto, Toronto, Canada

**Keywords:** subjective continuity, self-continuity, temporal processing, biographical disruptions, I-self, autobiographical memory, episodic memory, autonoetic consciousness

## Abstract

The sense that ourself is continuous over time is a ubiquitous human experience. Although episodic autobiographical memory (AM) makes some contribution to one's subjective sense of self-continuity—also known as I-self continuity or phenomenological continuity—there is growing evidence that individuals with memory disorders report intact phenomenological continuity. These observations suggest that additional processes must also contribute to phenomenological continuity. This systematic search and critical review aimed to identify candidate bodily and psychological processes underlying phenomenological continuity, with a focus on processes beyond AM. A systematic search of three databases (PsycINFO, PubMed and Web of Science) yielded 153 publications that provided evidence of several processes contributing to phenomenological continuity: interoception, embodiment, temporality, a stable life trajectory and nostalgia. Alongside episodic AM, these processes provide multiple sources of constancy integrated across differing timescales. In addition, we identified several processes that indirectly influence the saliency and expression of phenomenological continuity, such as ageing, socio-cultural factors and narrative processes. These findings extend existing models of self-continuity, providing a more comprehensive overview of the processes that, along with memory, underpin phenomenological continuity.

This article is part of the discussion meeting issue ‘From being to identity: analysis and synthesis of the self’.

## 1. Introduction

The question of how we know and feel that we are the same continuous person over time has been pondered for millennia. Yet the underpinnings of this phenomenon, known as diachronic unity, remain unclear. Previous work has linked diachronic unity to two forms of self-continuity based on William James’ [1] distinction of the I-self or ‘self-as-experiencer’ that actively reflects upon the Me-self or ‘self-as-experienced’ [2–4]. On this view, Me-self continuity, also known as semantic continuity, corresponds to the temporally extended representation of the self, including the self-concept and life story. In contrast, I-self continuity, or phenomenological continuity,^1^ refers to a moment-to-moment phenomenon of experiencing the self as extended in time that comprises both the ‘warmth and intimacy’ that James ascribes to the pre-reflective I-self [1] as well as the higher-order experiences of self-awareness and self-reflection [3]. Some have viewed this continuous I-self as an inner essence that owns and experiences all of our thoughts, perceptions and feelings (e.g. [12]). These inner states may change from one moment to the next, and the people and places that make up our lives may come and go, yet the ‘experiencer’ persists. Others have instead conceived of phenomenological continuity as arising from the flow of consciousness itself (e.g. [13]). It is the relations and resemblances between the inner experiential states that produce a sense of sameness over time—be these temporal contiguity, causal connections or the fact that all experiences that occur within me are infused with the same ‘warmth and intimacy’ [1] that is unique to me and makes them mine. The question of whether an ‘experiencer’ exists or whether, as the latter view supposes, it is illusory is beyond the scope of this article (see [14] for a recent treatment of this question). The current review focuses on the processes that underpin phenomenological continuity in light of apparently paradoxical findings emerging in the literature.

One influential theory is that phenomenological continuity depends heavily on episodic autobiographical memory (AM) and episodic future thinking [15,16] that enable the conscious (p)re-experience of the self at particular moments in the past and future [3,9]. Characteristic of this type of remembering is autonoetic consciousness that places past, present and future selves on a subjective timeline [17]. As such, a substantial portion of the theoretical and empirical literature on phenomenological continuity has focused on episodic AM as a key underlying mechanism [2,3,7,8,18–24]. At its most extreme, memory loss in amnesic individuals, such as patient H.M., results in a profound sense of the self being stuck in the present rather than extending over time [25,26]. Moreover, other studies report that less severe episodic AM deficits, such as those evident in Alzheimer's disease [27,28] and Asperger syndrome [29], are also associated with some degree of phenomenological discontinuity.

Nevertheless, contradictory evidence is emerging, with findings that people with significant loss of episodic AM, owing to Alzheimer's disease and episodic amnesia following hippocampal damage, are just as likely as healthy controls to report that they feel like they are fundamentally the same person as they were in the past [10,30–33]. If these individuals have a reduced capacity to re-experience their past selves via episodic AM, what then underlies their phenomenological continuity? Residual ‘islands’ of episodic AM may give some of these individuals sufficient linkages to their past selves. However, processes aside from episodic AM likely provide other means for phenomenological continuity.

Aspects of interoception and embodiment that contribute to the pre-reflective I-self in the present moment [6,34–36] also have temporal features that may be important to phenomenological continuity [37–39]. For instance, the ongoing experience of inhabiting the same body [35] or the constancy of bodily signals [40] may be a source of phenomenological continuity even when memory is impaired [2]. Interestingly, individuals with memory loss exhibit preservation of the embodied self, including bodily movements, gestures and habits [35,41–44]. We have recently suggested that the implicit reinstatement of bodily representations, states and experiences may give rise to phenomenological continuity even when memories of these states and experiences cannot be remembered [2]. In contrast, the experience of certain impactful events can create rifts or ‘biographical disruptions’ in the expected course of one's life [7] that may result in phenomenological discontinuity.

The objective of this systematic search and critical review is to assess the theoretical and empirical evidence that these potential processes—and any others that emerge through our search—support phenomenological continuity. It is worth considering the various ways in which phenomenological continuity has been operationalized in empirical research. One set of methods focuses on implicit, unconscious or embodied aspects of phenomenological continuity using qualitative approaches [45–47] or behavioural paradigms [48,49]. Other methods probe the reflective aspects of phenomenological continuity by measuring the perceived similarity of, or psychological connection between, the present self and the self in the past or future (e.g. continuous identity Venn diagram [50,51], self-continuity index [52] and self-continuity interview [53,54]). Neuroimaging (e.g. functional magnetic resonance imaging (fMRI) and electroencephalography (EEG); [55,56]) and neuropsychological (e.g. [10,29,31,57]) approaches have also been leveraged to shine light on the neural correlates of phenomenological (dis)continuity. Any given measure only taps into one aspect of phenomenological continuity, so only illuminating part of this complex construct. In this systematic search and review, we identified and synthesized findings generated by these different methodologies with available theoretical perspectives with the aim of developing a more comprehensive explanation of phenomenological continuity that accounts for its preservation in the face of significant memory loss.

## 2. Methods

### (a) Inclusion and exclusion criteria

Articles that covered at least one potential process underlying phenomenological continuity^2^ were considered. To be exhaustive, we included articles irrespective of population, publication date, design or data type. Exclusion criteria were as follows: not examining phenomenological continuity (e.g. present-moment I-self and forms of Me-self continuity); examining phenomenological continuity as underlying another mental process; conference abstracts, commentaries, editorials, book reviews and corrections; and articles where the main text was not in English.

### (b) Search strategy

Our search strategy involved two approaches. Our primary method was systematic searches of three databases—PsycINFO, PubMed and Web of Science—conducted on 6 and 7 June 2023. The search query included terms related to self-continuity (table 1); to cast a wide net, these terms captured all forms of self-continuity, including those usually associated with the Me-self (e.g. narrative continuity). A set of excluded terms was used to streamline the results. Our secondary method, pearl-growing using citations from the database results and PubMed alerts, identified additional papers published online up until 4 September 2024.^3^

**Table 1.** Search terms and Boolean operators used for literature database searches. Boolean operators are in capital case; asterisk indicates wildcard.

terms related to self-continuity	NOT	terms related to other (excluded) self-related constructs
‘phenomenological continuity’ OR ‘subjective continuity’ OR ‘self-continuity’ OR ‘semantic continuity’ OR ‘narrative continuity’ OR ‘temporal continuity’ OR ‘diachronic unity’		self-report* OR self-assess* OR self-evaluat* OR self-injur* OR self-disgust or self-administrat* OR self-manage* OR self-regulat* OR self-esteem OR self-control OR self-understanding OR self-disclosure OR self-interest OR self-limit* OR self-replicat* OR self-complement* OR self-adjuvant* OR self-rat* OR self-assembl* OR self-organi* OR self-select* OR self-sampl* OR self-associat*

### (c) Article selection

As presented in the PRISMA diagram (figure 1), the systematic searches yielded 2025 records that were submitted to two levels of screening by O.M.D. with input from D.R.A. and L.J.T. First-level screening focused on titles and abstracts; duplicate results were removed, and inclusion/exclusion criteria applied. Second-level screening was of full texts; if available in English, these were read and exclusion criteria applied as necessary. Included papers were then categorized according to publication type and the candidate process(es) covered. Publication lists from each screening step are publicly available (https://osf.io/qz8bm).

**Figure 1.**
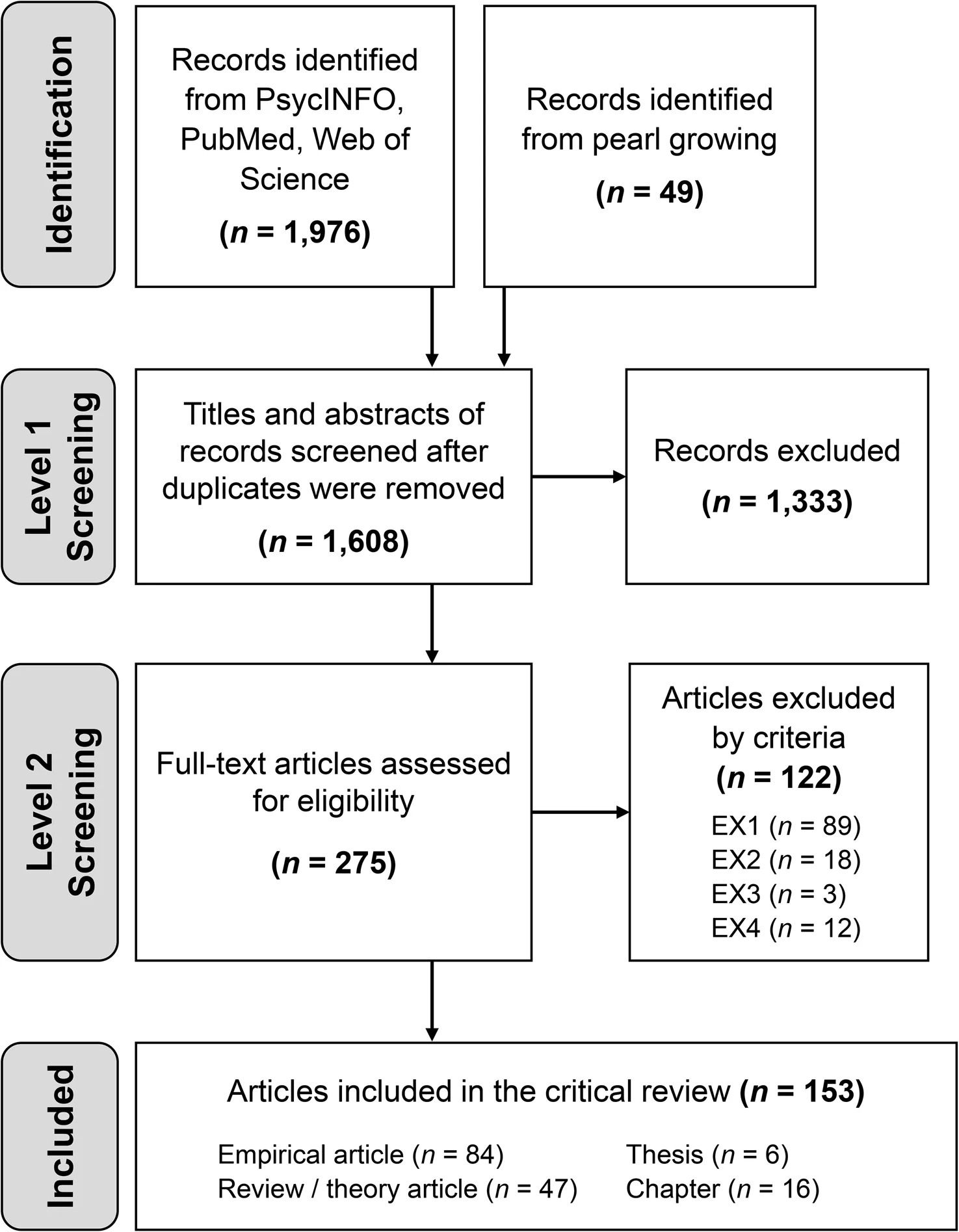
PRISMA flow diagram outlining the selection process for article selection and screening. EX, exclusion criteria; EX1, focus on present-moment I-self or semantic/narrative continuity; EX2, focus on phenomenological continuity as underlying another process; EX3, excluded publication type (conference/symposium abstract, book review); EX4, full text in language other than English.

## 3. Results

Our systematic search yielded 153 publications between 1987 and 2025 that cover six processes that potentially contribute to phenomenological continuity: AM (*n* = 45), interoception (*n* = 13), embodiment (*n* = 41), temporality (*n* = 17), life trajectory stability (*n* = 56) and nostalgia (*n* = 25; figure 2; for a list of papers in each category, see supplemental material, table S1). As the current focus was on identifying non-mnemonic processes underlying phenomenological continuity, we restricted our synthesis to all identified processes except episodic AM. Note that not all 153 papers are cited below; the full list of references is available in section S2 in the supplemental material.

**Figure 2.**
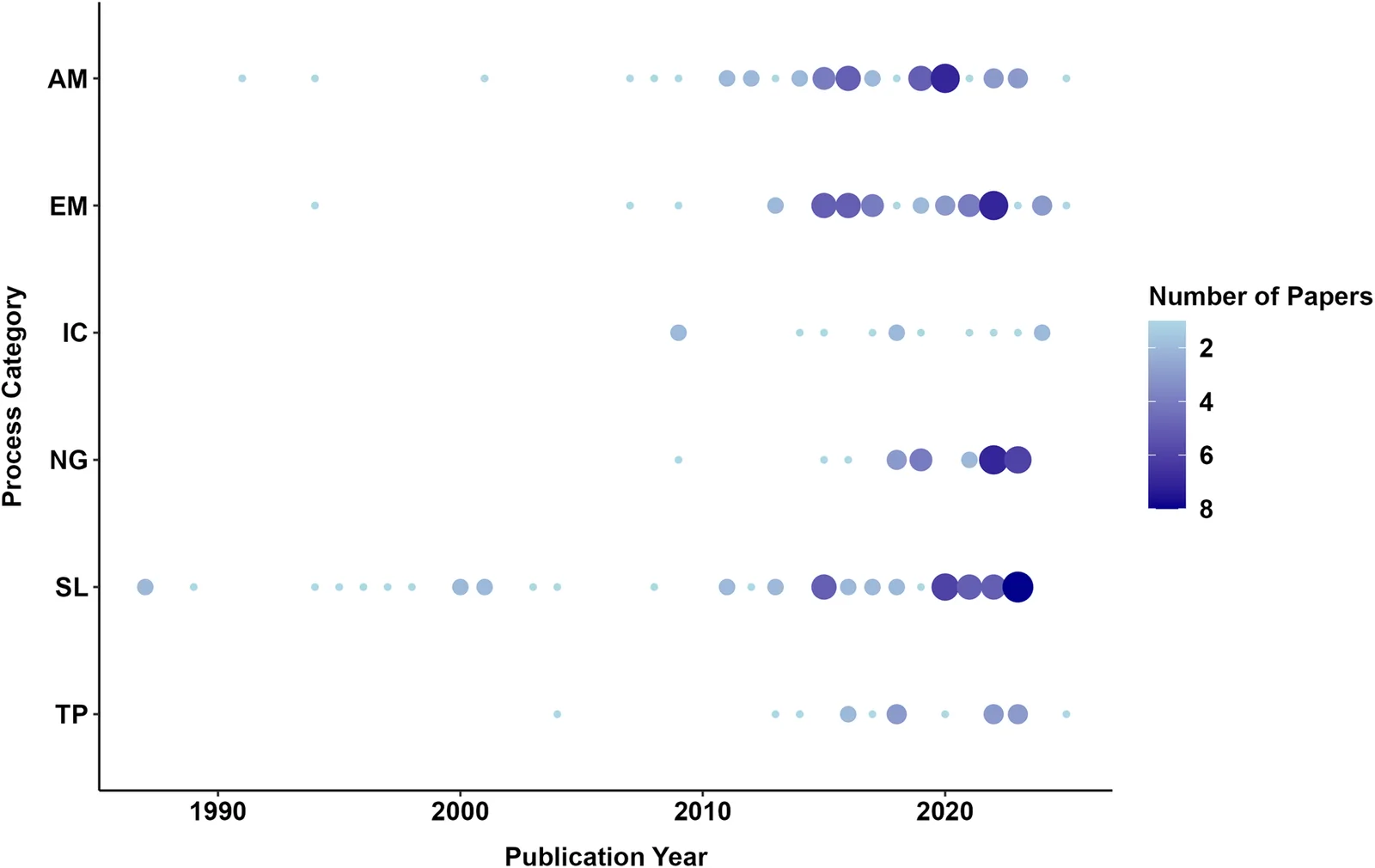
Bubble plot illustrating the publication trends of articles by process category. The size of each bubble represents the number of papers published each year between 1987 and 2025 in a given process category. Some papers examined multiple processes, so the total number of papers represented in the plot (*n* = 197) exceeds the number included in the critical review (*n* = 153). AM, autobiographical memory; EM, embodiment; IC, interoception; NG, nostalgia; SL, stable life trajectory; TP, temporality.

### (a) Interoception

A small set of 13 papers, mostly theoretical, described how interoception—the perception of internal stimuli, such as heart rate, pain and affective states—could play an important role in phenomenological continuity at the level of physiological systems. Available evidence from electrophysiological, neuroimaging and lesion studies suggest that the insula integrates interoceptive signals not only in a particular moment but across an extended period of time surrounding that moment [38,39] and integrates this representation with other self-relevant information [55]. In these accounts, the temporal extension of interoceptive feelings is on the order of (sub-)seconds, which, some argue, may be too short to underpin phenomenological continuity [28,58]. Nevertheless, we surmise that this extension of the I-self into the very near past and future may set a vital foundation for phenomenological continuity across longer timescales.

Indeed, visceral monitoring occurs not only over short timescales (e.g. heartbeat) but also over longer ones (e.g. digestion). Multiple review papers examining the role of the body in phenomenological continuity have proposed that integrating interoceptive information across several timescales may, in itself, be a source of phenomenological continuity [35,59,60], possibly at an implicit, unconscious level. Additionally, with their relative invariance and continuously cyclic nature that occur within their own nested time cycle [61], visceral and bodily signals often appear relatively static to the experiencer, providing a bodily constancy that can persist not only across days or years but even across one's lifetime [35,60]. The regulation of bodily systems through change (i.e. interoceptive active inference) results in a strong subjective expectation of bodily stability that may contribute to ‘change-blindness’ for the self [62]. It is possible that apparent stability of one's internal milieu over long timescales could be fundamental to phenomenological continuity. These authors also draw on reports from studies that observed that the present-moment I-self is altered with insula dysfunction and other conditions affecting interoception (e.g. [63–66]) to speculate that interoceptive deficits may affect phenomenological continuity [27,67]. However, our search did not reveal any studies testing this hypothesis directly, and this would be a fruitful avenue for future research.

### (b) Embodiment

Forty-one results referred to the idea that the existence of the lived body across one's life plays a pivotal role in phenomenological continuity ([35,59], but see [23]). One aspect of embodiment is the sense of agency or perceived control over one's body. Agency depends on the dynamic integration of experience over time, which has been hypothesized to occur in conjunction with a precise timing system—albeit also on the order of (sub-)seconds—making this another candidate process we found was theorized to support phenomenological continuity. Specifically, a combination of empirical evidence and theoretical work shows incoming multisensory signals, such as actions and thoughts, are compared with expectations of future signals derived from prior experience via predictive coding mechanisms; when these signals match, an experience is identified as qualitatively mine [48] and in turn belonging to the I-self [61].

Much of the literature linking embodiment to phenomenological continuity focuses on implicit forms of body memory that encapsulate all bodily constancies, including habits, physical capabilities and the entire history of the body's lived experience [35,59]. For instance, many authors contend that habits can stabilize the I-self over time, given their slow-changing nature and repetition across many contexts and time points [2,7,20,29,35,68]. A couple of qualitative studies suggested that habits result in the recall of meaningful sensations (e.g. baking cookies and lighting candles) and so could also act as vehicles for phenomenological continuity [69,70].

Another form of body memory, Rilkean memories, represents pre-reflective bodily-based actions and dispositions that accompany movements and experiences. It has been theorized that Rilkean memories are stored within the body independently from other kinds of memory, including episodic and procedural memories of the experience [35,71]. For example, a qualitative study that examined the lived experiences of people living with dementia provides evidence that access to embodied memory in the form of re-enacting bodily-oriented activities likely enables a pre-reflective form of phenomenological continuity that is not dependent on episodic memory [72]. An interesting claim, based on an interpretation of the work by Winnicott, an influential British psychoanalyst, is that very early experiences, such as movement *in utero*, may provide essential precursors to the development of a temporally extended I-self [73]. Furthermore, at a more explicit level, an anthropological study observed that individuals recovering from eating disorders appear to leverage memories of bodily states and somatic practices as a way to bridge the gap between their temporally distant I-selves, thereby integrating the lived experiences of one's past disorder and present recovery [45].

A number of papers linked phenomenological continuity with the updating of one's body representation. Although some researchers are beginning to consider the role of pre-experiencing one's future body—either via imagination or age-morphing technology [49,74–76]—most focus on responses to physical changes (e.g. [46,77]). For example, a case study of a woman with a sternotomy scar following open-heart surgery observed that she initially experienced diminished phenomenological continuity, manifesting as a sense of defamiliarization and not ‘being-at-home’ within her body. However, her sense of phenomenological continuity was restored through the integration of her scar within her embodied representation via affective touch and meaning-making [46]. The perceived effect of changes to body representation on phenomenological continuity can vary. A review paper drawing on published case reports of individuals with deep brain stimulators explains that, following implantation, some people report restorative effects on phenomenological continuity, whereas others describe detrimental effects. The authors argue that these differences reflect how well these individuals have integrated the neurostimulation device into their body representation [78]. Another study with a group of stroke survivors found many exhibited a high degree of phenomenological continuity, suggesting that experiencing embodied physical changes does not inevitably result in a sense of being fundamentally different [79].

Yet, the disruption of other forms of embodiment may be enough to produce a diminished sense of phenomenological continuity. Multiple review papers [80–84] and an empirical study [85] suggest that psychotic and dissociative symptoms experienced in schizophrenia, bipolar disorder and dissociative identity disorder profoundly alter many aspects of embodiment, including agency, body ownership and lived experience, resulting in a fragmented, unstable and weakened sense of phenomenological continuity. Findings regarding whether temporary alterations of embodied experience, such as during meditation, can result in fluctuations of phenomenological continuity are mixed. Two studies found that meditation affects the continuity of the I-self [56,58], possibly because the combination of heightened bodily attention, a perceived cessation of time and changes in physiological activity reduces the salience of the boundary between self and non-self. In contrast, a third study reported no association between trait mindfulness and phenomenological continuity [86]. The mixed findings presented by these studies suggest that changes to one's body representation can, in many cases, be eventually integrated into one's I-self without permanently disrupting phenomenological continuity. However, the health conditions examined in this literature vary in terms of the severity and the specific aspects of embodiment impacted, likely accounting for some of the variability observed in how such changes influence phenomenological continuity across conditions.

### (c) Temporality

Seventeen papers addressed the role of what we refer to collectively as temporality in phenomenological continuity, including inner time processing, the perceived passage of time and one's orientation in subjective time (i.e. towards the past versus future). A number of theories focus on temporal properties of brain signals as providing a means for self-related information—including experiential aspects—to be propagated across time. For instance, Revach and Salti [87] propose that predictive coding mechanisms prioritize the regularities of experience at the expense of fluctuations or deviations. Through the continuity of experience, a history of thoughts, feelings and perceptions accumulates in coarse-grained representations that smooth the transitions between states over time. They link this neural integration between discrete moments of time to phenomenological continuity. In a recent paper [2], we make a similar proposal that the history dependence of neural signals in the default mode network enables the integration of overlapping trajectories of both slow- and fast-changing experiences (see ‘Discussion’ for a more detailed discussion, including a related theory from Northoff and colleagues [11,88,89]). Fuchs [35,82] also advances a neuro-phenomenological account that applies philosophical arguments from Husserl, Heidegger and Merleau-Ponty to understanding the effect of psychopathology on phenomenological continuity. Fuchs and colleagues propose that the processing of time operates on two interrelated levels: an implicit, pre-reflective flow of lived time and an explicit, reflective awareness of temporal succession. According to this neuro-phenomenological account, there are two prerequisites for implicit time processing, both of which relate to the I-self: (i) the constitutive synthesis of inner experience, referring to the combination of one's sense of what is to come next, what is occurring in a particular moment and what has just immediately happened—corresponding to Husserl's concepts of protentions, presentations and retentions; and (ii) conation-affective dynamics, encompassing the basic energetic or motivational states, such as drives and urges that imbue one's subjective experiences with a ‘sense of aliveness’ [82]. Relatedly, converging neuroscientific evidence suggests that the temporal overlap of different aspects of one's experience gives rise to a sense of continuous subjective experience, and this may be supported by the temporal properties of brain activity [2]. Moreover, extraction of regularities in information related to the I-self across time can be modelled and organized into hierarchical systems that give rise to both phenomenological and semantic forms of continuity [87].

Several review articles linked abnormal temporal processing to disruptions of phenomenological continuity in psychiatric conditions, including depression [59,80,82], bipolar disorder [59,81,82], borderline personality disorder [81] and schizophrenia [57,80,82,90], albeit with some differences between conditions. For example, depression is often associated with a subjective slowing or cessation of time, thought to be related to feeling as if one is stuck in the present yet preoccupied with the past [80] or to a form of hyper-embodiment in which one feels stuck within the confines of their own body and isolated from the passing experiences of the external world [59,82]. People with bipolar disorder report experiencing time as slowing down during depressive episodes, but as speeding up during manic episodes [59,81,82], whereas people with borderline personality tend to experience time as fragmented, rapidly switching from one present to the next [81]. These authors have argued that these alterations of temporality are the source of reduced phenomenological continuity in mood disorders. Schizophrenia, on the other hand, is marked by a global disconnect between the immediate past, present and anticipated future that Fuchs has proposed could be owing to impairments in both the constitutive synthesis of inner experience and conation-affective dynamics aspects of implicit time processing [59,82].

Age-related differences in time perception may help explain empirical findings that, relative to younger adults, older adults generally exhibit a stronger sense of phenomenological continuity [91–94]. One explanation is that older adults typically perceive time as passing more quickly, leading to a compressed sense of time that may be experienced as an ‘extended present’. This temporal compression reduces the perceived distance between past, present and future selves [91,92], thus heightening phenomenological continuity. Cross-cultural differences in phenomenological continuity may also relate to temporality. For instance, Ji *et al.* [91,95] found that relative to Westerners who tend to be present-focused, East Asian participants showed stronger connections to both the past and future that were associated with stronger phenomenological continuity—perhaps again via a compression of perceived distances on one's subjective timeline (but see [96]). Further, other studies have found that Eastern cultures place more value on past events and traditions, whereas Western cultures are more future-focused and emphasize progress—and phenomenological continuity mirrors this pattern, with Westerners showing greater future asymmetry (i.e. higher future continuity relative to past continuity) than individuals from Eastern cultures [97].

### (d) Stability of life trajectory

A stable life trajectory shapes and informs the lived experience of the I-self over time [7,20], and events that drastically change this trajectory were identified as detrimental to phenomenological continuity in 56 papers, which included many empirical studies and a few reviews about the effects of biographical disruptions on phenomenological continuity. Diagnosis of a health condition, from dementia to depression [30,98], is a common biographical disruption that can throw into disarray the life one had envisaged for oneself, either through unexpected challenges, the support needed from others, loss of employment or changes in one's goals. Transitions, such as adolescence, socio-cultural upheaval (e.g. the First Nations self-determination movement [53,99,100]) and global events (e.g. the COVID-19 pandemic [101,102]), have been found to have similar effects. Other examples include existential anxiety [103], stressors associated with belonging to a minority group (e.g. discrimination [104]) and significant life events such as relationships ending or changes in occupations [7,8,20,52,105]. When these events constitute biographical disruptions, individuals report perceiving a sense of derailment: a subjective feeling at the level of the I-self that one's life is on an unexpected or ‘off-course’ trajectory [98].

Particular biographical disruptions may also impact the stability of other processes underpinning phenomenological continuity, such as embodiment [7,20]. For instance, the development of a child within the mother's body during pregnancy produces a unique intercorporeal relationship that temporarily fuses one's I-self with another being—an alteration to the mother's body representation that is soon reversed by childbirth, as observed in a qualitative study that interviewed mothers who gave birth prematurely [47]. Life changes can also disrupt the embodied sense of ‘being at home’ in particular places, that is the qualitative feeling of familiarity with spaces and cultures that are experienced as one's ‘own’ [106]. A number of articles, both primary studies and reviews, suggested that profound changes in sense of place, whether provoked by migration or displacement ([106–114]; but see [115]) or transitioning from deployment to civilian life [116], may especially threaten phenomenological continuity. Our sense of being at home initially develops through secure attachments to our carers in early life and, in turn, influences the development of the I-self over time [111]. We surmise that the sense of ‘homelessness’ is one reason why biographical disruptions and adversity occurring in childhood can be particularly detrimental for phenomenological continuity [105,117].

There are several ways in which phenomenological continuity can be maintained or restored when one's life trajectory is disrupted. One is through the incorporation of changes as part of one's I-self, which the dialogical approach [118] provides a framework for understanding. On this account, the I-self takes different forms or ‘I-positions’ across different spaces, social interactions and therefore across time (e.g. ‘I-as-my body’ and ‘I-as-mother’). As Salgado *et al.* [118] argue, ‘at every moment, every person occupies a contextualized ‘position’ regarding the world … the self develops as a chain of positions that succeed each other in time’ (pp. 327–328). Although instability is inherent to this ‘micro’ level as I-positions change from moment to moment, these can be integrated as manifestations of the same I-self by tracing sequences of I-positions [118]. Further unification is thought to occur through self-related dialogue, such as internal thoughts or social conversations that invite self-reflection (e.g. psychotherapy), by which individuals relate I-positions to each other over longer time spans, identify repetitions of and similarities across I-positions and develop meta-I-positions that afford high-level perspectives on the entire life course [118]. Two recent studies provide evidence that during migration [107] and the transition to motherhood [47], the emergence of meta-positions enabled individuals to reconcile and incorporate changes to the I-self and ultimately maintain or even enhance their sense of phenomenological continuity. Moreover, while some aspects of the I-self may be experienced as ‘off-course’ during a biographical disruption, others remain on a continuous life trajectory. Additionally, continued interactions with meaningful activities, people and possessions [29,107,117,119]—including culturally shared practices, symbols and traditions [53,120–122]—may counteract threats to phenomenological continuity. For instance, a study on hoarding reported that individuals with Asperger syndrome use their collection of objects as a form of external scaffolding to counteract the negative impacts of AM deficits on their phenomenological continuity [29].

Threats to phenomenological continuity can also be mitigated through the endorsement of beliefs and explanations about self-continuity, such as understanding ourselves as beings who are capable of change [113], and that, despite changes, we remain fundamentally the same at our core [53,99,120,123]. Change can also be framed as one arc within the broader life story. Although these narrative processes are integral to the Me-self, empirical studies have found that individuals can help repair the discontinuity experienced in the I-self by actively integrating change [7,108].

Culture and age shape how individuals maintain phenomenological continuity in the face of biographical disruptions. Studies show explanatory strategies and beliefs about self-continuity differ across cultures [53,124,125], which we surmise may be called upon to varying extents depending on socio-cultural background and lived experience. Moreover, age may bring greater capacity to navigate biographical disruptions. Indeed, despite the challenges to sense of self that accompany ageing, including the loss of independent living, the death of loved ones and declines in the bodily self, several studies report evidence that phenomenological continuity is stronger with older age [76,92,105,119].

### (e) Nostalgia

The role of nostalgia in phenomenological continuity emerged in 25 papers. Nostalgia is an ambivalent affective state combining yearning, wistfulness and bittersweetness with a positivity for people or places from ‘times gone by’ [126]. Although fundamentally self-relevant, nostalgia is also social, typically experienced by people reminiscing, and cultural, with rituals or traditions often the focus of this yearning [70,112]. While reminiscing can trigger AM recall of specific events and lifetime periods, nostalgia is a distinct process characterized by its emotional and social nature. Most studies report a positive relationship between nostalgia and phenomenological continuity [19,127,128], evident regardless of culture [102,126].

Nostalgia involves making links between one's past, present and even future, resulting in a broader outlook on life that emphasizes commonalities, which, in turn, could allow one to view one's self as connected over time at an experiential, subjective level [129]. Additionally, nostalgizing provides affective connections between the present and temporally distant selves. A similar relationship has been reported between phenomenological continuity and awe [130], suggesting affective experiences that reach over time, rather than nostalgia itself, may afford a source of phenomenological continuity. Both nostalgia and awe likely evoke autonoetic consciousness, enabling a person to remember what it *felt* like to be oneself in the past, without necessarily recalling specific episodic details. An alternative view is that the relationship between nostalgia and phenomenological continuity is mediated by other factors, including social connectedness and belongingness [126,128], ostracism (i.e. the opposite of social connectedness [131]), narrative processes [132,133] and holistic thinking [129].

## 4. Discussion

The emergence of evidence that people with severe episodic AM impairments still report a fundamental feeling of being the same person over time (e.g. [10,30,31]) requires that models of phenomenological continuity extend beyond AM. In line with our hypotheses, the results identified contributions of bodily-oriented and psychological processes, specifically interoception, embodiment, temporality, stability of one's life trajectory and nostalgia. Although some of these processes—namely embodiment, life trajectory stability and nostalgia—draw on memory to some extent, these forms of memory are distinct from episodic AM. The set of processes identified here, taken in conjunction with episodic AM, provides a more complete mechanistic explanation of phenomenological continuity and allows for a level of redundancy so that only in the most extreme of cases is phenomenological continuity lost. These findings indicate that our original model that focused on AM [2,3] should be updated to incorporate this broader range of processes underpinning phenomenological continuity.

### (a) Sources of constancy and reconciliation of change

Each of the identified processes offers self-stability and constancy, instrumental in the development and maintenance of phenomenological continuity. Interoception, for example, incorporates visceral and bodily signals, continuously cyclical and relatively invariant, which provide stability across long timescales from days to one's entire lifetime [35,60]. Similarly, the storage and reinstatement of many forms of embodied memories, including Rilkean memories and habits, provide a profound sense of inhabiting the same body over time (e.g. [35]). Whether or not particular types of embodied memories—such as those frequently accessed (e.g. habits) or those more central to the I-self—have a privileged role in phenomenological continuity is unclear. One's sense of place, or ‘being at home’ [106,110], as well as continuity of interpersonal relationships, may be elements of ‘the thread of a lived life’ [14, p. 95] that maintains phenomenological continuity in times of biographical disruptions.

Embodiment is intertwined with interoception, consistent with arguments that the two processes evolved alongside each other [134]; interoceptive states are experienced by, and emerge in response to, a body that is engaging with the world with a sense of agentic control. However, in line with influential frameworks of pre-reflective self, the internal milieu also includes perceptions, actions, thoughts, and other cognitions and feelings [135–137], with researchers such as Damasio [135], Panksepp [136] and Asma and Gabriel [137] emphasizing emotion and affect as particularly central. We found that the recurrence of these feeling states across time, via both interoception [38,39] and nostalgia [112,126,128], provides another important source of constancy. Relatedly, many authors [17], including ourselves [3], have argued that (p)re-experiencing these feeling states as extended over time at a reflective level is uniquely underpinned by episodic AM and future thinking. Our findings dovetail with an emerging perspective that these autonoetic states can be mediated by non-episodic forms of AM [138] and by non-mnemonic mechanisms such as embodiment and interoception [139].

Some mechanisms can account for change. Despite being in constant flux, the ongoing regulation of physiological systems gives a somewhat illusory perception of relative stasis—a form of self-change-blindness [62], with predictive coding for future bodily and feeling states playing a major role in this illusion [38,139]. Conversely, recognizing changes in the I-self can also foster phenomenological continuity by prompting comparison across two points of time. Thus, both ignoring and attending to change can inform phenomenological continuity, albeit in different ways. Additionally, changes in the I-self may initiate adaptation that provides its own source of continuity, such as updating one's body representation [46] or I-positions [107,118]. Fluctuations in the I-self can also be regulated through the capacity for taking meta-perspectives on the commonalities of one's I-self across time and space [47,118] or the extraction of regularities in self-experience that are then propagated across time [87,139].

Perhaps unsurprisingly, temporality emerged as critical to understanding the continuity of the I-self over time. As put by Tafarodi, ‘We cannot apprehend ourselves outside of time … temporality is inherent to being’ [140, p. 33]. Although as Prescott & Dominey [139] remind us, continuity does not require a calibrated mechanism for measuring time; all that is needed is the ability to distinguish between now and then. Interestingly, some theories actually posit a central role of interoception in how time is registered in the brain, stemming from evidence that the anterior insula cortex houses ‘an endogenous time base’ [38, p. 1932]. In particular, Wittman [141] proposes that successions of bodily signals are stored as pulses in the insula that accumulate when attention is directed to time, which informs duration judgements and, in turn, forms the basis of subjective time. For most, if not all, of the processes examined here, continuity is afforded by some form of recurrence or temporal integration, so it is interesting to note that many of them exist at multiple timescales. Interoception, for example, exists both on the order of seconds [38,39] and longer timescales, ranging from hours, days, to one's entire lifetime [35,60]. Conation-affective dynamics (e.g. motivational states such as urges and drives) also lie at the intersection of interoception and temporality [82]. Furthermore, embodied memories can reference momentary bodily experiences but arguably also capture the body's lived history from birth until death [35,59].

Taken together, many of the mechanisms we have identified in this review as underpinning phenomenological continuity span across or occur at differing timescales. We speculate that the intersection of these timescales could be a key mechanism for phenomenological continuity [2]. According to the principles of hierarchical integration (e.g. [142,143]), transient forms of continuity operating below the level of awareness (e.g. agency) are nested within consciously accessible continuity signals, and rapid fluctuations of experience are incorporated with both persisting signals and gradual changes. Neuroimaging evidence suggests that brain regions are organized according to the timescales of the signals they process [142,144], and that the temporal structure of brain signals may underpin aspects of the I-self, including its continuity [37,145]. Indeed, a number of studies conducted by Northoff and colleagues provide compelling evidence of a relationship between self-related processing and the particular temporal properties of neural activity in the default mode network (for comprehensive reviews and discussion of their spatiotemporal neuroscience framework, see [11,88,89]). Their findings suggest that continuity is associated with the long autocorrelation window of activity in these brain regions that arguably reflects the neural recurrence or temporal ‘expansion’ of self-relevant information over time [145], akin to the brain's ‘memory’ for its processing history. Moreover, the temporal structure of these brain regions is scale-free, with patterns echoing across infra-slow to fast timescales [11]. Northoff's framework is a ‘basis model’ of the self in that it posits that this multiscale integration is a fundamental property of the brain that underpins even pre-reflective aspects of self and is therefore independent of perception, cognition and affect [146]. The findings of the critical review, as interpreted here, complement this basis model by elucidating similarly structured temporal dynamics in both pre-reflective and reflective forms of continuity, with both bodily signals and higher-order self-reflective thoughts repeating across multiple timescales.

### (b) Modulators of phenomenological continuity

The results of this critical review also revealed processes that shape the saliency and expression of phenomenological continuity. A particularly noteworthy finding was that despite reductions in episodic AM, older adults report stronger phenomenological continuity than younger adults. Although linked to alterations in temporality, such as a compressed sense of time [91,92], we suggest that age-related asymmetries in the amount of life yet-to-be or already lived may also modulate other processes underpinning phenomenological continuity. With a greater accumulation of lived experience and more opportunities for the recurrence of interoceptive states and habits over much longer time expanses, older adults should necessarily have stronger self-continuity signals with their past selves than younger adults. In contrast, although younger adults can experience phenomenological continuity over long expanses with their future selves, by definition, this self-continuity is not based on lived experience, and therefore, the quality—or at least strength—of future phenomenological continuity likely differs from older adults. Additionally, psychosocial maturation likely alters the salience of one's phenomenological continuity. The focus during younger adulthood on goal-pursuit, new roles and identities, and personal growth [147] may de-emphasize phenomenological continuity. In contrast, goal orientation shifts towards maintenance and stability [147], which likely enhances phenomenological continuity. Further, the increasing need to reflect on, and make sense of, one's life trajectory takes prominence in later life [148], which has been related to age-related increases in nostalgia [149].

Culture also shapes the way we perceive our phenomenological continuity [140]. Cultural heritage, including long-standing traditions, customs and extended family histories, provides a form of continuity operating on a timescale beyond one's own lifetime that may heighten one's own perceived phenomenological continuity. Additionally, culture can provide resilience in the face of personal challenges [53] and when threatened or destabilized by colonization or migration, there is usually a striving to protect the group's continuity [150]. Cultural retention is not always possible, however; in such cases, individual members may be more vulnerable to phenomenological discontinuity and poorer psychological outcomes [120,123]. There are cross-cultural differences in how time is perceived and subjectively experienced [140], and we suggest, as have others (e.g. [140]), that phenomenological continuity is shaped by culturally shared conceptions of time. Other cultural frames, such as norms of personhood and ‘life scripts’ of what a typical life should look like, also influence phenomenological continuity. For instance, in Western culture, there is an implicit theory of development—that we grow from experience and that events can change us [151]. Even so, there is also a shared cultural understanding that when someone claims to be a ‘new’ or ‘changed’ person, they are speaking metaphorically. In fact, it is considered abnormal or a crisis of identity for someone to fundamentally feel like they are no longer the same person [84]. Schechtman [14] observes that when someone does become a wholly different person owing to extreme experiences of trauma or neurological conditions that markedly alter personality, both the changed person and those around them are disconcerted or even frightened, in large part because of the strong-held belief that a person should be the same self over time.

Narrative processes, such as autobiographical reasoning used to construct a coherent life story, might also be an important modulator. Although narrative forms of continuity are typically considered distinct from phenomenological continuity in that they rely on the content of the Me-self, the possibility of interactions between semantic or narrative forms of continuity and phenomenological continuity has been raised in the literature [7,20], particularly in the context of biographical disruptions. Narrative processes can be used to link distant elements of one's life with the current I-self in ways that bridge discontinuity. When disruptions and changes are actively embedded within the overarching structure of the life story, this compensation can re-enable phenomenological continuity [7]. Narrative processes can support and augment the gradual integration of bodily changes within one's self-representation to maintain phenomenological continuity following a health condition (e.g. [46]). We propose that in some cases the integration of physiological changes may need to be accompanied by narrative work to fully restore phenomenological continuity. This point is illustrated by the case of a woman where restoration of her phenomenological continuity following sternotomy surgery required meaning-making to fully integrate her scar within her embodied representation [46]. Furthermore, one's subjective outlook is central to narrating one's life story; the I-self is the narrator of the life story, actively processing life experiences, gaining insights and making meaning [152,153]. At any moment, the experience of the I-self is understood through the lens of our current ‘working’ narrative of the self; in this way, narratives are a form of predictive processing that serves to organize patterns of experience and reduce uncertainty that are integral to phenomenological continuity [139].

### (c) Phenomenological discontinuity

An important question is whether our I-self can ever truly be discontinuous. Some argue discontinuity is inconceivable; the experiencer is fundamental to having experience [33,115]. By this view, diachronic disunity must arise from changes to the Me-self. An alternative view is that the convergence of the multiple processes identified in this review (and perhaps others we have not identified) provides a redundancy that keeps phenomenological continuity ever-present. All of these processes likely have to be absent in order not to experience any phenomenological continuity at all.

However, phenomenological continuity may exist on a gradient in which perceived self-continuity is a matter of degree [78]. The diminishment of certain processes may have greater effects on phenomenological continuity than others. Although future research is required to clarify how each process differentially contributes to phenomenological continuity, we speculate that pre-reflective bodily-oriented processes in particular, such as interoception and embodiment, play a particularly fundamental role—not only in maintaining phenomenological continuity but also in its earliest development (e.g. *in utero*, [62,73]). Studies involving populations with impairments in the identified processes could further clarify whether disturbances of certain (or combinations of) processes are more detrimental for phenomenological continuity. It is also possible that the qualitative nature of phenomenological continuity could differ depending on which processes are being leveraged. For example, individuals with episodic AM deficits may demonstrate a different ‘flavour’ of phenomenological continuity than individuals with intact AM. Additionally, while the modulators may explain group differences, there may also be individual differences in the processes not captured by the results. Why is it that not everyone who experiences disturbances in the identified processes feels a sense of phenomenological discontinuity, at least for some period of time? Although there could be additional complex factors at play, from early life experiences to well-being, the threshold of perceiving discontinuity at the level of the I-self may vary across individuals.

### (d) Heterogeneity of articles

A key strength—but also a challenge—of this review was its interdisciplinary scope, integrating philosophical and theoretical approaches with empirical evidence from a variety of methods tapping both pre-reflective and reflective aspects of phenomenological continuity. Despite this, the results coalesced around the six identified processes, and there was a strong convergence across sources in how these processes are thought to support phenomenological continuity. One reason for this could be because of many of the empirical and theoretical frameworks being influenced by and grounded in key philosophical arguments. Nevertheless, as we have commented previously [3], the proliferation of self-related nomenclature within and across these literatures remains a challenge. Notably, many empirical studies do not theoretically situate their methods for measuring continuity, with it not always readily apparent whether they are probing pre-reflective or reflective aspects of phenomenological continuity—or whether they are even examining continuity or the I-self at all. The field would be strengthened by work to better map both existing and new measures to theories of continuity.

## 5. Conclusion

To our knowledge, this review is the first to systematically identify and synthesize the range of processes underlying phenomenological continuity. The results revealed that, alongside AM, multiple processes, including interoception, embodiment, temporality, a stable life trajectory and nostalgia, provide various sources of constancy at both short and long timescales. We suggest that the hierarchical integration of both pre-reflective and reflective experience across differing timescales could be key to phenomenological continuity, and that the reliance on multiple processes may augment continuity as well as protect against discontinuity. We hope that this more comprehensive explanation of phenomenological continuity will be a useful foundation for future empirical research into how it is we perceive ourselves as continuous over time.

## Supplementary Material



## Data Availability

The data supporting this article are publicly available on the Open Science Framework [154]. Supplementary material is available online [155].

## Footnotes

^1^
In the same way that many terms have been used to describe the I-self, including the minimal self [5], proto-self and core-self [6], the temporally extended I-self has been referred to as self-continuity [7,8], subjective continuity [4] and phenomenological continuity [3]. Following our previous work [2,3,9,10], here we use the term phenomenological continuity. Our framework proposes that phenomenological continuity, together with the temporally extended Me-self (or semantic and narrative forms of continuity), contributes to diachronic unity—the perception of the self as the same over time despite changes. Diachronic unity is comparable to the concept of ‘personal identity’ as described by Northoff [11].

^2^
Phenomenological continuity was broadly defined as the temporal extension of aspects of self that are pre-reflective, subjective, phenomenological or experiential, as opposed to the known aspects of self, such as the self-concept and narratives.

^3^
Note that articles with a publication date of 2025 were available online in 2024.

## Theme

One contribution of 17 to a discussion meeting issue ‘From being to identity: analysis and synthesis of the self’.

## References

[1] James W . 1890 The principles of psychology. New York, NY: Henry Holt and Company.

[2] Addis DR , Davis OM, Tippett LJ. 2025Autobiographical memory and the integrated self. In Encyclopedia of the human brain, 2nd edn (ed. Grafman JH), pp. 793–806. Amsterdam, The Netherlands: Elsevier. (doi:10.1016/B978-0-12-820480-1.00185-6)

[3] Prebble SC , Addis DR, Tippett LJ. 2013 Autobiographical memory and sense of self. Psychol. Bull. 139, 815–840. (doi:10.1037/a0030146) 23025923

[4] Strikwerda-Brown C , Grilli MD, Andrews-Hanna J, Irish M. 2019 ‘All is not lost’—rethinking the nature of memory and the self in dementia. Ageing Res. Rev. 54, 100932. (doi:10.1016/j.arr.2019.100932) PMC6726574 31238174

[5] Gallagher S . 2000 Philosophical conceptions of the self: implications for cognitive science. Trends Cogn. Sci. 4, 14–21. (doi:10.1016/S1364-6613(99)01417-5) 10637618

[6] Damasio A . 1999 The feeling of what happens: body and emotion in the making of consciousness. New York, NY: Harcourt Brace and Co.

[7] Habermas T , Köber C. 2015 Autobiographical reasoning in life narratives buffers the effect of biographical disruptions on the sense of self-continuity. Memory 23, 664–674. (doi:10.1080/09658211.2014.920885) 24912017

[8] Sedikides C , Hong EK, Wildschut T. 2023 Self-continuity. Annu. Rev. Psychol. 74, 333–361. (doi:10.1146/annurev-psych-032420-032236) 35961040

[9] Addis DR , Tippett LJ. 2008The contributions of autobiographical memory to the content and continuity of identity: a social-cognitive neuroscience approach. In Self continuity: individual and collective perspectives (ed. Sani F), pp. 71–86. Abingdon, UK: Psychology Press.

[10] Tippett LJ , Prebble SC, Addis DR. 2018 The persistence of the self over time in mild cognitive impairment and Alzheimer's disease. Front. Psychol. 9, 94. (doi:10.3389/fpsyg.2018.00094) PMC5826309 29515473

[11] Northoff G . 2017 Personal identity and cortical midline structure (CMS): do temporal features of CMS neural activity transform into ‘self-continuity’ ? Psychol. Inq. 28, 122–131. (doi:10.1080/1047840X.2017.1337396)

[12] Descartes R . [1637] 1960 Discourse on method and meditations. New York, NY: The Liberal Arts Press.

[13] Hume D . [1739] 2000 A treatise of human nature. Oxford, UK: Oxford University Press.

[14] Schechtman M . 2024 The self: a very short introduction. Oxford, UK: Oxford University Press.

[15] Atance CM , O'Neill DK. 2001 Episodic future thinking. Trends Cogn. Sci. 5, 533–539. (doi:10.1016/s1364-6613(00)01804-0) 11728911

[16] Schacter DL , Addis DR. 2007 The cognitive neuroscience of constructive memory: remembering the past and imagining the future. Phil. Trans. R. Soc. B 362, 773–786. (doi:10.1098/rstb.2007.2087) PMC2429996 17395575

[17] Tulving E . 1985 How many memory systems are there? Am. Psychol. 40, 385–398. (doi:10.1037/0003-066x.40.4.385)

[18] Allé MC , D'Argembeau A, Schneider P, Potheegadoo J, Coutelle R, Danion J-M, Berna F. 2016 Self-continuity across time in schizophrenia: an exploration of phenomenological and narrative continuity in the past and future. Compr. Psychiatry 69, 53–61. (doi:10.1016/j.comppsych.2016.05.001) 27423345

[19] Areni CS , Momeni M, Reynolds N. 2022 Ontological insecurity, nostalgia, and social media: viewing YouTube videos of old TV commercials reestablishes continuity of the self over time. Psychol. Popul. Media 11, 227–236. (doi:10.1037/ppm0000352)

[20] Habermas T , Köber C. 2015Autobiographical reasoning is constitutive for narrative identity: the role of the life story for personal continuity. In The Oxford handbook of identity development (eds KC McLean, M Syed), pp. 149–165. Oxford, UK: Oxford University Press.

[21] Sani F . 2008Introduction and overview. In Self contininuity: individual and collective perspectives (ed. Sani F), pp. 1–12. Abingdon, UK: Psychology Press.

[22] Sokol Y , Conroy AK, Weingartner KM. 2017 The cognitive underpinnings of continuous identity: higher episodic memory recall and lower heuristic usage predicts highest levels of self-continuity. Identity 17, 84–95. (doi:10.1080/15283488.2017.1303384)

[23] Taylor JG . 2007 On the neurodynamics of the creation of consciousness. Cognit. Neurodynam. 1, 97–118. (doi:10.1007/s11571-006-9011-8) PMC2267666 19003506

[24] Liao HW . 2017 Being me over time: the self-continuity function of autobiographical memory in adulthood. PhD thesis, University of Florida, United States.

[25] Postle BR . 2016The hippocampus, memory, and consciousness. In The neurology of consciousness, 2nd edn. (eds Laureys S, Gosseries O, Tononi G), pp. 349–363. San Diego, CA: Academic Press. (doi:10.1016/B978-0-12-800948-2.00021-2)

[26] Corkin S . 2013 Permanent present tense: the unforgettable life of amnesic patient H. M. New York, NY: Basic Books.

[27] Tisserand A , Philippi N, Botzung A, Blanc F. 2023 Me, myself and my insula: an oasis in the forefront of self-consciousness. Biology 12, 599. (doi:10.3390/biology12040599) PMC10135849 37106799

[28] Weiler M , Northoff G, Damasceno BP, Balthazar MLF. 2016 Self, cortical midline structures and the resting state: implications for Alzheimer's disease. Neurosci. Biobehav. Rev. 68, 245–255. (doi:10.1016/j.neubiorev.2016.05.028) 27235083

[29] Skirrow P , Jackson P, Perry E, Hare DJ. 2015 I collect therefore I am—autonoetic consciousness and hoarding in Asperger syndrome. Clin. Psychol. Psychother. 22, 278–284. (doi:10.1002/cpp.1889) 24615808

[30] Clare L , Martyr A, Morris RG, Tippett LJ. 2020 Discontinuity in the subjective experience of self among people with mild-to-moderate dementia is associated with poorer psychological health: findings from the IDEAL cohort. J. Alzheimer's Dis. 77, 127–138. (doi:10.3233/JAD-200407) PMC7592652 32804138

[31] Davis OM . 2020 Who am I: sense of self in individuals with sudden-onset episodic amnesia. Unpublished Master's thesis, University of Auckland, New Zealand.

[32] El Haj M , Allain P. 2020 Self-defining memories and their contribution to the sense of self in Alzheimer's disease. Curr. Alzheimer Res. 17, 508–516. (doi:10.2174/1567205017666200807184942) 32851943

[33] Klein SB . 2014 Sameness and the self: philosophical and psychological considerations. Front. Psychol. 5, 29. (doi:10.3389/fpsyg.2014.00029) PMC3905202 24523707

[34] Christoff K , Cosmelli D, Legrand D, Thompson E. 2011 Specifying the self for cognitive neuroscience. Trends Cogn. Sci. 15, 104–112. (doi:10.1016/j.tics.2011.01.001) 21288760

[35] Fuchs T . 2017 Self across time: the diachronic unity of bodily existence. Phenomenol. Cogn. Sci. 16, 291–315. (doi:10.1007/s11097-015-9449-4)

[36] Wehrle M . 2021 ‘Bodies (that) matter’: the role of habit formation for identity. Phenomenol. Cogn. Sci. 20, 365–386. (doi:10.1007/s11097-020-09703-0)

[37] Qin PM , Wang MX, Northoff G. 2020 Linking bodily, environmental and mental states in the self—a three-level model based on a meta-analysis. Neurosci. Biobehav. Rev. 115, 77–95. (doi:10.1016/j.neubiorev.2020.05.004) 32492474

[38] Craig ADB . 2009 Emotional moments across time: a possible neural basis for time perception in the anterior insula. Phil. Trans. R. Soc. Lond. B 364, 1933–1942. (doi:10.1098/rstb.2009.0008) PMC2685814 19487195

[39] Craig ADB . 2009 How do you feel—now? The anterior insula and human awareness. Nat. Rev. Neurosci. 10, 59–70. (doi:10.1038/nrn894) 19096369

[40] Davey CG , Harrison BJ. 2022 The self on its axis: a framework for understanding depression. Transl. Psychiatry 12, 1–9. (doi:10.1038/s41398-022-01790-8) PMC8766552 35042843

[41] Caddell LS , Clare L. 2010 The impact of dementia on self and identity: a systematic review. Clin. Psychol. Rev. 30, 113–126. (doi:10.1016/j.cpr.2009.10.003) 19896760

[42] Kontos PC . 2004 Ethnographic reflections on selfhood, embodiment and Alzheimer's disease. Ageing Soc. 24, 829–849. (doi:10.1017/S0144686X04002375)

[43] Kontos PC . 2003 ‘The painterly hand’: embodied consciousness and Alzheimer's disease. J. Aging Stud. 17, 151–170. (doi:10.1016/S0890-4065(03)00006-9)

[44] Scoville WB , Milner B. 1957 Loss of recent memory after bilateral hippocampal lesions. J. Neurol. Neurosurg. Psychiatry 20, 11–21. (doi:10.1136/jnnp.20.1.11) PMC497229 13406589

[45] Eli K . 2016 The body remembers: narrating embodied reconciliations of eating disorder and recovery. Anthropol. Med. 23, 71–85. (doi:10.1080/13648470.2015.1135786) 26982433

[46] Tomán E , Pintér JN, Hargitai R. 2024 The role of the lived body during the integration of the traumatic experience of the sternotomy scar: a case study. J. Eval. Clin. Pract. 30, 804–816. (doi:10.1111/jep.13925) 37723845

[47] Uriko K , Bartels I. 2023 Separation in unity: dialogical transformation of maternal bond. Integr. Psychol. Behav. Sci. 57, 590–606. (doi:10.1007/s12124-022-09688-2) 35412263

[48] Stern Y , Ben-Yehuda I, Koren D, Zaidel A, Salomon R. 2022 The dynamic boundaries of the self: serial dependence in the sense of agency. Cortex 152, 109–121. (doi:10.1016/j.cortex.2022.03.015) 35550935

[49] Lee AR . 2017 Psychological proximity to issues of the elderly: the role of age-morphing technology in campaigns for the elderly. Comput. Hum. Behav. 69, 311–323. (doi:10.1016/j.chb.2016.12.046)

[50] Ersner-Hershfield H , Garton MT, Ballard K, Samanez-Larkin GR, Knutson B. 2009 Don't stop thinking about tomorrow: individual differences in future self-continuity account for saving. Judgm. Decis. Mak. 4, 280–286. (doi:10.1017/S1930297500003855) PMC2747683 19774230

[51] Sokol Y , Eisenheim E. 2016 The relationship between continuous identity disturbances, negative mood, and suicidal ideation. Prim. Care Companion CNS Disord. 18, 1–11. (doi:10.4088/PCC.15m01824) PMC4874761 27247841

[52] Sedikides C , Wildschut T, Routledge C, Arndt J. 2015 Nostalgia counteracts self-discontinuity and restores self-continuity. Eur. J. Soc. Psychol. 45, 52–61. (doi:10.1002/ejsp.2073)

[53] Chandler MJ , Lalonde CE, Sokol BW, Hallett D, Marcia JE. 2003 Personal persistence, identity development, and suicide: a study of Native and Non-Native North American adolescents. Monogr. Soc. Res. Child Dev. 68, 1–138. (doi:10.1111/1540-5834.00246) 12951783

[54] Troll LE , Skaff MM. 1997 Perceived continuity of self in very old age. Psychol. Aging 12, 162–169. (doi:10.1037/0882-7974.12.1.162) 9100277

[55] Scalabrini A , Wolman A, Northoff G. 2021 The self and its right insula—differential topography and dynamic of right vs. left insula. Brain Sci. 11, 1312. (doi:10.3390/brainsci11101312) PMC8533814 34679377

[56] Fingelkurts AA , Fingelkurts AA, Kallio-Tamminen T. 2016 Long-term meditation training induced changes in the operational synchrony of default mode network modules during a resting state. Cogn. Proc. 17, 27–37. (doi:10.1007/s10339-015-0743-4) 26525051

[57] Lin YS , Hartwich P, Wolff A, Golesorkhi M, Northoff G. 2020 The self in art therapy—brain-based assessment of the drawing process. Med. Hypotheses 138, 109 596. (doi:10.1016/j.mehy.2020.109596) 32059158

[58] Droit-Volet S , Dambrun M. 2019 Awareness of the passage of time and self-consciousness: what do meditators report? PsyCh J. 8, 51–65. (doi:10.1002/pchj.270) 30740922

[59] Fuchs T . 2018 The cyclical time of the body and its relation to linear time. J. Conscious. Stud. 25, 47–65.

[60] Park HD , Blanke O. 2019 Coupling inner and outer body for self-consciousness. Trends Cogn. Sci. 23, 377–388. (doi:10.1016/j.tics.2019.02.002) 30826212

[61] Izadifar M . 2022 The neurobiological basis of the conundrum of self-continuity: a hypothesis. Front. Psychol. 13, 740542. (doi:10.3389/fpsyg.2022.740542) PMC9159515 35664197

[62] Seth AK , Tsakiris M. 2018 Being a beast machine: the somatic basis of selfhood. Trends Cogn. Sci. 22, 969–981. (doi:10.1016/j.tics.2018.08.008) 30224233

[63] Hu L , He H, Roberts N, Chen J, Yan G, Pu L, Song X, Luo C. 2023 Insular dysfunction of interoception in major depressive disorder: from the perspective of neuroimaging. Front. Psychiatry 14, 1273430. (doi:10.3389/fpsyt.2023.1273439) PMC10568471 37840807

[64] García-Cordero I *et al.* 2016 Feeling, learning from and being aware of inner states: interoceptive dimensions in neurodegeneration and stroke. Phil. Trans. R. Soc. B 371, 20160006. (doi:10.1098/rstb.2016.0006) PMC5062096 28080965

[65] Lucherini Angeletti L *et al.* 2022 Anorexia nervosa as a disorder of the subcortical–cortical interoceptive-self. Eat. Weight Disord. 27, 3063–3081. (doi:10.1007/s40519-022-01510-7) PMC9803759 36355249

[66] Paulus MP , Stein MB. 2010 Interoception in anxiety and depression. Brain Struct. Funct. 214, 451–463. (doi:10.1007/s00429-010-0258-9) PMC2886901 20490545

[67] Monti A , Porciello G, Panasiti MS, Aglioti SM. 2022 The inside of me: interoceptive constraints on the concept of self in neuroscience and clinical psychology. Psychol. Res. 86, 2468–2477. (doi:10.1007/s00426-021-01477-7) PMC9674731 34050431

[68] Northoff G . 2020Habits and self: a temporal view. In Habits: pragmatist approaches from cognitive science, neuroscience, and social theory (eds F Caruana, I Testa), pp. 58–78. Cambridge, UK: Cambridge University Press. (doi:10.1017/9781108682312.003)

[69] Low KEY . 2013 Olfactive frames of remembering: theorizing self, senses and society. Sociol. Rev. 61, 688–708. (doi:10.1111/1467-954X.12078)

[70] Waskul DD , Vannini P, Wilson J. 2009 The aroma of recollection: olfaction, nostalgia, and the shaping of the sensuous self. Sens. Soc. 4, 5–22. (doi:10.2752/174589309X388546)

[71] Leyva A . 2022 Embodied movement consciousness. Phenomenol. Cogn. Sci. 21, 161–180. (doi:10.1007/s11097-021-09792-5)

[72] Owen S , Page S, Ledingham K, Price S, Connell J, Quinn C, Clare L. 2024 Embodied leisure experiences of nature-based activities for people living with dementia. Dementia 23, 1081–1102. (doi:10.1177/14713012241262384) PMC11440788 39058935

[73] Elkins J . 2015 Motility, aggression, and the bodily I: an interpretation of Winnicott. Psychoanal. Q. 84, 943–973. (doi:10.1002/psaq.12044) 26443951

[74] Blouin-Hudon EMC , Pychyl TA. 2017 A mental imagery intervention to increase future self-continuity and reduce procrastination. Appl. Psychol. 66, 326–352. (doi:10.1111/apps.12088)

[75] Mertens ECA , Siezenga AM, Tettero T, van Gelder JL. 2022 A future orientation intervention delivered through a smartphone application and virtual reality: study protocol for a randomized controlled trial. BMC Psychol. 10, 315. (doi:10.1186/s40359-022-01025-x) PMC9768900 36539846

[76] Tausen BM , Csordas A, MacRae CN. 2020 The mental landscape of imagining life beyond the current life span: implications for construal and self-continuity. Innov. Aging 4, 1–16. (doi:10.1093/geroni/igaa013) PMC7447858 32864477

[77] Godoy-Casasbuenas N , Cadena-Camargo Y, Rodríguez N, de Vries E, Yaron G. 2024 ‘You can be cured, but cancer never leaves you behind’: an interdisciplinary approach into the embodied cancer experiences among adult Colombian childhood/adolescent cancer survivors. J. Psychosoc. Oncol. 42, 859–874. (doi:10.1080/07347332.2024.2345112) 38698529

[78] Nyholm S , O'Neill E. 2016 Deep brain stimulation, continuity over time, and the true self. Camb. Q. Healthc. Ethics 25, 647–658. (doi:10.1017/S0963180116000372) 27634716

[79] Walker S . 2017 Stroke, self, identity and post-stroke psychological distress. Unpublished PhD thesis, University of Auckland, New Zealand.

[80] Bizzari V . 2023 Which kind of body in ‘mental’ pathologies? Phenomenological insights on the nature of the disrupted self. J. Med. Phil. 48, 116–127. (doi:10.1093/jmp/jhad008) 37078730

[81] Borda JP . 2016 Self over time: another difference between borderline personality disorder and bipolar disorder. J. Eval. Clin. Pract. 22, 603–607. (doi:10.1111/jep.12550) 27144989

[82] Fuchs T . 2013 Temporality and psychopathology. Phenomenol. Cogn. Sci. 12, 75–104. (doi:10.1007/s11097-010-9189-4)

[83] Putnam FW . 1994Dissociation and disturbances of self. In Rochester symposium on developmental psychopathology: disorders and dysfunctions of the self (eds D Cicchetti, Toth SL), pp. 251–265. New York, NY: University of Rochester Press.

[84] Rashed MA . 2021 An approach to the boundary problem: mental health activism and the limits of recognition. Phil. Psychiatry Psychol. 28, 297–313. (doi:10.1353/ppp.2021.0047)

[85] Dorahy MJ , Huntjens RJC, Marsh RJ, Johnson B, Fox K, Middleton W. 2021 The sense of self over time: assessing diachronicity in dissociative identity disorder, psychosis and healthy comparison groups. Front. Psychol. 12, 620063. (doi:10.3389/fpsyg.2021.620063) PMC7902028 33633645

[86] Naisani Samani M , Busseri MA. 2019 Examining the link between mindfulness and temporal perspective. Mindfulness 10, 2647–2660. (doi:10.1007/s12671-019-01240-5)

[87] Revach D , Salti M. 2022 Consciousness as the temporal propagation of information. Front. Syst. Neurosci. 16, 759683. (doi:10.3389/fnsys.2022.759683) PMC8984189 35401129

[88] Northoff G , Ventura B. 2025 Bridging the gap of brain and experience—converging neurophenomenology with spatiotemporal neuroscience. Neurosci. Biobehav. Rev. 173, 106 139. (doi:10.1016/j.neubiorev.2025.106139) 40204159

[89] Northoff G , Buccellato A, Ventura B. 2025 The default mode network and inner time consciousness. Curr. Opin. Behav. Sci. 63, 101524. (doi:10.1016/j.cobeha.2025.101524)

[90] Martin B , Wittmann M, Franck N, Cermolacce M, Berna F, Giersch A. 2014 Temporal structure of consciousness and minimal self in schizophrenia. Front. Psychol. 5, 1175. (doi:10.3389/fpsyg.2014.01175) PMC4212287 25400597

[91] Ji LJ , Imtiaz F, Su Y, Zhang Z, Bowie AC, Chang B. 2022 Culture, aging, self-continuity, and life satisfaction. J. Happiness Stud. 23, 3843–3864. (doi:10.1007/s10902-022-00568-5) PMC9514170 36187718

[92] Löckenhoff CE , Rutt JL. 2017 Age differences in self-continuity: converging evidence and directions for future research. Gerontologist 57, 396–408. (doi:10.1093/geront/gnx010) PMC5434491 28520940

[93] Lu Y , Gerstorf D, Löckenhoff CE. 2023 Age differences in self-continuity in Germany and the United States: the role of temporal direction, temporal distance, and demographics. J. Gerontol. Ser. B 78, 841–846. (doi:10.1093/geronb/gbad002) PMC10174281 36617791

[94] Rutt JL , Löckenhoff CE. 2016 Age patterns in mental representations of time: underlying constructs and relevant covariates. Exp. Aging Res. 42, 289–306. (doi:10.1080/0361073X.2016.1156975) PMC4830914 27070047

[95] Ji LJ , Hong EK, Guo T, Zhang Z, Su Y, Li Y. 2018 Culture, psychological proximity to the past and future, and self-continuity. Eur. J. Soc. Psychol. 49, 735–747. (doi:10.1002/ejsp.2544)

[96] Tafarodi RW , Lo C, Yamaguchi S, Lee WWS, Katsura H. 2004 The inner self in three countries. J. Cross-Cult. Psychol. 35, 97–117. (doi:10.1177/0022022103260462)

[97] Callizo-Romero C *et al.* 2022 Does time extend asymmetrically into the past and the future? A multitask crosscultural study. Lang. Cogn. 14, 275–302. (doi:10.1017/langcog.2022.5)

[98] Ratner K , Burrow AL. 2021 Derailment within the landscape of psychopathology. Curr. Opin. Psychol. 41, 21–27. (doi:10.1016/j.copsyc.2021.01.009) 33662865

[99] Ball L , Chandler MJ. 1989 Identity formation in suicidal and nonsuicidal youth: the role of self-continuity. Dev. Psychopathol. 1, 257–275. (doi:10.1017/S0954579400000444)

[100] Chandler MJ . 1994 Self-continuity in suicidal and nonsuicidal adolescents. New Dir. Child Adolesc. Dev. 1994, 55–70. (doi:10.1002/cd.23219946406) 7936350

[101] Cho H , Li P, Chen A. 2024 Nostalgia, social media, and subjective wellbeing: the dualistic and conditional effects of nostalgia during the COVID-19 pandemic. Health Commun. 39, 507–517. (doi:10.1080/10410236.2023.2170723) 36727655

[102] Lee-Won RJ , Lee EJ, Lee JA. 2023 Nostalgic social media use and psychological well-being during the COVID-19 pandemic. Cyberpsychol. Behav. Soc. Netw. 26, 90–97. (doi:10.1089/cyber.2022.0281) 36723518

[103] Berenskötter F . 2020 Anxiety, time, and agency. Int. Theory 12, 273–290. (doi:10.1017/S1752971920000111)

[104] Martin-Storey A , Recchia HE, Santo JB. 2021 Self-continuity moderates the association between sexual-minority status based discrimination and depressive symptoms. J. Homosex. 68, 2075–2096. (doi:10.1080/00918369.2020.1733350) 32130077

[105] Lampraki C , Spini D, Jopp DS. 2023 Life span development of self-continuity in late adulthood: the impact of chronological age, loss of partner and aversive childhood events. Self Identity 22, 669–688. (doi:10.1080/15298868.2022.2157869)

[106] Märtsin M , Mahmoud HW. 2012Never at home? Migrants between societies. In The Oxford handbook of culture and psychology (ed. Valsiner J), pp. 730–745. Oxford: Oxford University Press. (doi:10.1093/oxfordhb/9780195396430.013.0035)

[107] Bourke K , de Abreu G, Rathbone CJ. 2019 ‘I'm just who I am’: self-continuity and the dialogical self in a study of migrants. J. Construct. Psychol. 32, 274–291. (doi:10.1080/10720537.2018.1488227)

[108] Camia C , Zafar R. 2021 Autobiographical meaning making protects the sense of self-continuity past forced migration. Front. Psychol. 12, 618 343. (doi:10.3389/fpsyg.2021.618343) PMC7959802 33732188

[109] de la Mata-Benítez ML , Español A, Matías-García JA, Lojo M, Villar-Toribio C. 2024 Repairing the breach: identity narratives of a Latin American woman in Andalusia. Cult. Psychol. 30, 150–172. (doi:10.1177/1354067X231160237)

[110] Dowd A . 2020 Displacement trauma: complex states of personal, collective and intergenerational fragmentation and their intergenerational transmission. J. Anal. Psychol. 65, 300–324. (doi:10.1111/1468-5922.12588) 32170748

[111] Hill J . 1996 At home in the world. J. Anal. Psychol. 41, 575–598. (doi:10.1111/j.1465-5922.1996.00575.x)

[112] Sedikides C , Wildschut T. 2022 Nostalgia across cultures. J. Pac. Rim Psychol. 16, 1–16. (doi:10.1177/18344909221091649)

[113] Sedikides C , Wildschut T, Grouzet F. 2018 On the temporal navigation of selfhood: the role of self-continuity. Self Identity 17, 255–258. (doi:10.1080/15298868.2017.1391115)

[114] Zou X , Petkanopoulou K. 2023 Nostalgia and acculturation. Curr. Opin. Psychol. 49, 101553. (doi:10.1016/j.copsyc.2022.101553) 36696865

[115] Jacard SM . 2001 The impact of immigration on an individual's sense of self. Unpublished PhD thesis, Massachusetts School of Professional Psychology.

[116] Levin C , Nenninger S, Freundlich D, Glatt S, Sokol Y. 2023 How future self-continuity mediates the impact of job loss on negative mental health outcomes among transitioning veterans. Mil. Psychol. 36, 491–503. (doi:10.1080/08995605.2023.2215697) PMC11407375 37252891

[117] Ward H . 2011 Continuities and discontinuities: issues concerning the establishment of a persistent sense of self amongst care leavers. Child. Youth. Serv. Rev. 33, 2512–2518. (doi:10.1016/j.childyouth.2011.08.028)

[118] Salgado J , Cunha C, Bento T. 2013 Positioning microanalysis: studying the self through the exploration of dialogical processes. Integr. Psychol. Behav. Sci. 47, 325–353. (doi:10.1007/s12124-013-9238-y) 23797867

[119] Zittoun T , Grossen M, Tarrago Salamin F. 2021 Creating new spheres of experience in the transition to a nursing home. Learn. Cult. Soc. Int. 28, 100458. (doi:10.1016/j.lcsi.2020.100458)

[120] Chandler MJ , Proulx T. 2008Personal persistence and persistent peoples: continuities in the lives of individual and whole cultural communities. In Self continuity: individual and collective perspectives (ed. Sani F), pp. 213–226. Abingdon, UK: Psychology Press.

[121] Mellor A , Cedar Child and Family Services, Cloutier D. 2020 Becoming self-in-relation: coming of age as a pathway towards wellness for urban Indigenous youth in care. First Peoples Child Fam. Rev. 15, 1–22. (doi:10.7202/1080807ar)

[122] Syed M , Fish J. 2018 Revisiting Erik Erikson's legacy on culture, race, and ethnicity. Identity 18, 274–283. (doi:10.1080/15283488.2018.1523729)

[123] Chandler MJ , Lalonde CE. 1998 Cultural continuity as a hedge against suicide in Canada's first nations. Transcult. Psychiatry 35, 191–219. (doi:10.1177/136346159803500202)

[124] Dunlop WL , Walker LJ. 2015 Cross-cultural variability in self-continuity warranting strategies. J. Lang. Soc. Psychol. 34, 300–315. (doi:10.1177/0261927X14555873)

[125] Santo JB , da Cunha J, Saldarriaga LM, Recchia HE, Martin-Storey A, Stella-Lopez L, Carmago G, Bukowski WM. 2013 Measurement invariance of self-continuity strategies: comparisons of early adolescents from Brazil, Canada and Colombia. Eur. J. Dev. Psychol. 10, 518–525. (doi:10.1080/17405629.2012.707313)

[126] Sedikides C *et al.* 2016 Nostalgia fosters self-continuity: uncovering the mechanism (social connectedness) and consequence (eudaimonic well-being). Emotion 16, 524–539. (doi:10.1037/emo0000136) 26751632

[127] Layous K , Kurtz JL, Wildschut T, Sedikides C. 2021 The effect of a multi-week nostalgia intervention on well-being: mechanisms and moderation. Emotion 22, 1952–1968. (doi:10.1037/emo0000817) 34591502

[128] Sedikides C , Wildschut T. 2018 Finding meaning in nostalgia. Rev. Gen. Psychol. 22, 48–61. (doi:10.1037/gpr0000109)

[129] Hong EK , Sedikides C, Wildschut T. 2021 Nostalgia strengthens global self-continuity through holistic thinking. Cogn. Emot. 35, 730–737. (doi:10.1080/02699931.2020.1862064) 33356840

[130] Pan X , Jiang T. 2022 Awe fosters global self-continuity: the mediating effect of global processing and narrative. Emotion 23, 1618–1632. (doi:10.1037/emo0001187) 36355671

[131] Jiang T , Chen Z, Wang S, Hou Y. 2021 Ostracism disrupts self-continuity. Personal. Soc. Psychol. Bull. 47, 1390–1400. (doi:10.1177/0146167220974496) 33272111

[132] Dai Y , Li Q, Zhou H, Jiang T. 2023 Nostalgia and online autobiography: implications for global self-continuity and psychological well-being. J. Happiness Stud. 24, 2747–2763. (doi:10.1007/s10902-023-00701-y)

[133] Hong EK , Sedikides C, Wildschut T. 2022 How does nostalgia conduce to global self-continuity? The roles of identity narrative, associative links, and stability. Personal. Soc. Psychol. Bull. 48, 735–749. (doi:10.1177/01461672211024889) PMC9066684 34151655

[134] Feinberg TE , Mallatt JM. 2016 The ancient origins of consciousness: how the brain created experience. Cambrisge, MA: MIT Press.

[135] Damasio A . 2010 Self comes to mind: constructing the conscious brain. New York, NY: Pantheon Books.

[136] Panksepp J . 1998 The periconscious substrates of consciousness: affective states and the evolutionary origins of the self. J. Consciousness Stud. 5, 566–582.

[137] Asma ST , Gabriel R. 2019 The emotional mind: the affective roots of culture and cognition. Cambridge, MA: Harvard University Press.

[138] Addis DR , Szpunar KK. 2024 Beyond the episodic–semantic continuum: the multidimensional model of mental representations. Phil. Trans. R. Soc. B 379, 20230408. (doi:10.1098/rstb.2023.0408) PMC11449204 39278248

[139] Prescott TJ , Dominey PF. 2024 Synthesizing the temporal self: robotic models of episodic and autobiographical memory. Phil. Trans. R. Soc. B 379, 20230415. (doi:10.1098/rstb.2023.0415) PMC11523108 39278252

[140] Tafarodi RW . 2008Toward a cultural phenomenology of personal identity. In Self continuity: individual and collective perspectives (ed. Sani F), pp. 27–42. Abingdon, UK: Psychology Press.

[141] Wittmann M . 2015 Modulations of the experience of self and time. Conscious. Cogn. 38, 172–181. (doi:10.1016/j.concog.2015.06.008) 26121958

[142] Hasson U . 2025 Uncovering a timescale hierarchy by studying the brain in a natural context. J. Neurosci. 45, e2368242025. (doi:10.1523/jneurosci.2368-24.2025) PMC11924986 40107727

[143] Singhal I , Srinivasan N. 2021 Time and time again: a multi-scale hierarchical framework for time-consciousness and timing of cognition. Neurosci. Consciousness 2021, niab020. (doi:10.1093/nc/niab020) PMC8358708 34394957

[144] Kiebel SJ , Daunizeau J, Friston KJ. 2008 A hierarchy of time-scales and the brain. PLoS Comput. Biol. 4, e1000209. (doi:10.1371/journal.pcbi.1000209) PMC2568860 19008936

[145] Huang Z , Obara N, Davis H, Pokorny J, Northoff G. 2016 The temporal structure of resting-state brain activity in the medial prefrontal cortex predicts self-consciousness. Neuropsychologia 82, 161–170. (doi:10.1016/j.neuropsychologia.2016.01.025) 26805557

[146] Northoff G . 2016 Is the self a higher-order or fundamental function of the brain? The ‘basis model of self-specificity’ and its encoding by the brain's spontaneous activity. Cogn. Neurosci. 7, 203–222. (doi:10.1080/17588928.2015.1111868) 26505808

[147] Bühler JL , Weidmann R, Nikitin J, Grob A. 2019 A closer look at life goals across adulthood: applying a developmental perspective to content, dynamics, and outcomes of goal importance and goal attainability. Eur. J. Personal. 33, 359–384. (doi:10.1002/per.2194)

[148] Erikson EH . 1950 Childhood and society. New York, NY: W W Norton & Co.

[149] Turner JR , Stanley JT. 2021 Holding on to pieces of the past: daily reports of nostalgia in a life-span sample. Emotion 21, 951–961. (doi:10.1037/emo0000980) 33779191

[150] Smeekes A , Verkuyten M. 2015 The presence of the past: identity continuity and group dynamics. Eur. Rev. Soc. Psychol. 26, 162–202. (doi:10.1080/10463283.2015.1112653)

[151] Wilson AE , Ross M. 2001 From chump to champ: people's appraisals of their earlier and present selves. J. Pers. Soc. Psychol. 80, 572–584. (doi:10.1037/0022-3514.80.4.572) 11316222

[152] Velleman JD . 2006The self as narrator. In Self to self: selected essays. Cambridge, UK: Cambridge University Press.

[153] McAdams DP . 1996 Personality, modernity, and the storied self: a contemporary framework for studying persons. Psychol. Inq. 7, 295–321. (doi:10.1207/s15327965pli0704_1)

[154] Davis OM , Tippett LJ, Addis DR. 2025 Towards a comprehensive understanding of the processes underlying phenomenological continuity. (doi:10.17605/OSF.IO/QZ8BM)

[155] Davis OM , Tippett LJ, Addis DR. 2026 Supplementary material from: Towards a comprehensive understanding of the processes underlying phenomenological continuity. Figshare. (doi:10.6084/m9.figshare.c.8718631)

