# Supplementary material for: Towards a comprehensive understanding of the processes underlying phenomenological continuity
Source: Philos Trans R Soc Lond B Biol Sci. 2026 Nov 10;381(1962):20250162. doi: 10.1098/rstb.2025.0162 (PMC13750419; doi:10.1098/rstb.2025.0162)
Supplement: Supplementary file 1 [file rstb.2025.0162.zip › rstb.2025.0162/rstb20250162_si_001.docx]

**Supplemental Materials**

**Towards a Comprehensive Understanding of the Processes Underlying Phenomenological Continuity**

Odysse M. Davis, Lynette J. Tippett, and Donna Rose Addis

**Table S1**

*Categorization of final set of articles based on candidate process*

**Autobiographical memory**

Addis et al. (2025); Allé et al. (2016); Areni et al. (2022) Arzy et al. (2011); Blouin-Hudon et al. (2016); Bluck & Alea (2009); Bluck & Alea (2011); Caci et al. (2019); Caci et al. (2020); Clare et al. (2020); Cory (2012); Dary & Lopez (2023); Davis (2020); El Haj & Allain (2020); El Haj et al. (2019); Grabowski & Broemer (2015); Habermas & Köber (2015a); Habermas & Köber (2015b); Harris et al. (2021); Hazan et al. (2019); Jiang et al. (2020); Jobson et al. (2019); Klein (2014); Lewin (1991); Liao & Bluck (2022); Liao et al. (2016); Liao (2017); Lind et al. (2020); Prebble et al. (2013); Putnam (1994); Raby & Clayton (2012); Sani (2008); Sedikides et al. (2023); Shen et al. (2022); Skirrow et al. (2015); Sokol et al. (2017); Sokol & Serper (2019); Tanguay et al. (2020); Taylor (2007); Tippett et al. (2018); Tisserand et al. (2023); Weiler et al. (2016); Wilson (2014); Wolf & Zimprich (2016); Zelazo & Sommerville (2001)

**Interoception**

Brinkers et al. (2024); Craig (2009a); Craig (2009b); Fuchs (2017); Fuchs (2018); Mensch (2015); Monti et al. (2022); Park & Blanke (2019); Park & Tallon-Baudry (2014); Parvizi-Wayne et al. (2024); Scalabrini et al. (2021); Seth & Tsakiris (2018); Tisserand et al. (2023)

**Embodiment**

Addis et al. (2025); Bizzarri (2023); Blouin-Hudon & Pychyl (2017); Blouin-Hudon et al. (2016); Borda (2016); Chrishima & Wilson (2021); Dorahy et al. (2021); Dowd (2020); Droit-Volet & Dambrun (2019); Eli (2016); Elkins (2015); Fingelkurts et al. (2016); Fuchs (2013); Fuchs (2017); Fuchs (2018); Ganschow et al. (2021); Godoy-Casabeun et al. (2024); Habermas & Köber (2015a); Habermas & Köber (2015b); Izadifar (2022); Lee (2017); Leyva (2022); Low (2013); Mensch (2015); Mertens, Siezenga, et al. (2022); Mertens, van der Schalk, et al. (2022); Monti et al. (2022);

Naisani Samani & Busseri (2019); Northoff (2020); Nyholm & O'Neill (2016); Owen et al. (2024); Putnam (1994); Rashed (2021); Shen et al. (2022); Skirrow et al. (2015); Stern et al. (2022); Tausen et al. (2020); Taylor (2007); Tomán et al. (2024); Walker (2017); Waskul et al. (2009)

**Temporality**

Addis et al. (2025); Bizzarri (2023); Borda (2016); Callizo-Romero et al. (2022); Fuchs (2013); Fuchs (2018); Ji et al. (2018); Ji et al. (2022); Lin et al. (2020); Löckenhoff & Rutt (2017); Lu et al. (2023); Martin et al. (2014); Revach & Salti (2022); Rutt & Löckenhoff (2016); Sedikides et al. (2018); Sedikides et al. (2023); Tafarodi et al. (2004)

**Stable life trajectory**

Ball & Chandler (1989); Berenskötter (2020); Bourke et al. (2019); Camia & Zafar (2021); Chandler (1994); Chandler (2000); Chandler (2001); Chandler et al. (1987); Chandler et al. (2000); Chandler et al. (2003); Chandler & Lalonde (1995); Chandler & Lalonde (1998); Chandler & Proulx (2008); Clare et al. (2020); de la Mata-Benitez et al. (2023); Dowd (2020); Dunlop & Walker (2015); Grandits (2020); Habermas & Köber (2015a); Habermas & Köber (2015b); Hill (1996); Izadifar (2022); Jacard (2001); Ji et al. (2022); Lalonde & Chandler (2004); Lampraki et al. (2023); Lee (2017); Lee-Won et al. (2023); Levin et al. (2023); Löckenhoff & Rutt (2017); Lu et al. (2023); Martin-Storey et al. (2021); Märtsin & Mahmoud (2012); Mellor et al. (2020); Mertens, van der Schalk, et al. (2022); Pachana & Bauchmeister (2021); Ratner & Burrow (2021); Ritchie et al. (2011); Rutt & Löckenhoff (2016); Salgado et al. (2013); Santo et al. (2013); Sedikides et al. (2023); Sedikides et al. (2015); Sedikides et al. (2016); Sedikides et al. (2018); Sedikides & Wildschut (2022); Shen et al. (2022); Skirrow et al. (2015); Smith (1987); Syed & Fish (2018); Tausen et al. (2020); Troll & Skaff (1997); Uriko & Bartels (2023); Ward (2011); Zittoun et al. (2021); Zou & Petkanopoulou (2023)

**Nostalgia**

Abakoumkin et al. (2019); Areni et al. (2022); Biskas et al. (2022); Cho et al. (2024); Dai et al. (2023); Evans (2022); Hong et al. (2021); Hong et al. (2022); Jiang et al. (2021); Juhl & Biskas (2023); Lasaleta & Loveland (2019); Layous et al. (2022); Lee-Won et al. (2023); Pan & Jiang (2022); Sedikides et al. (2015); Sedikides et al. (2016); Sedikides et al. (2018); Sedikides et al. (2023); Sedikides & Wildschut (2018); Sedikides & Wildschut (2019); Sedikides & Wildschut (2022); van Tilburg et al. (2019); Waskul et al. (2009); Wildschut et al. (2018); Zou & Petkanopoulou (2023)

*Note.* Articles can fall into more than one category.

**S2 References for final set of articles**

Abakoumkin, G., Hepper, E. G., Wildschut, T., & Sedikides, C. (2019). From nostalgia through social connectedness to self-continuity: Replication and extension. *Hellenic Journal of Psychology*, *16*, 127–144. [https://doi.org/10.26262/hjp.v16i2.789](https://doi.org/https://doi.org/10.26262/hjp.v16i2.789)

Addis, D. R., Davis, O. M., & Tippett, L. J. (2025). Autobiographical memory and the integrated self. In J. H. Grafman (Ed.), *Encyclopedia of the Human Brain* (2nd ed., pp. 793–806). Elsevier. <https://doi.org/10.1016/B978-0-12-820480-1.00185-6>

Allé, M. C., D’Argembeau, A., Schneider, P., Potheegadoo, J., Coutelle, R., Danion, J. M., & Berna, F. (2016). Self-continuity across time in schizophrenia: An exploration of phenomenological and narrative continuity in the past and future. *Comprehensive Psychiatry*, *69*, 53–61. <https://doi.org/10.1016/j.comppsych.2016.05.001>

Areni, C. S., Momeni, M., & Reynolds, N. (2022). Ontological insecurity, nostalgia, and social media: Viewing YouTube videos of old TV commercials reestablishes continuity of the self over time. *Psychology of Popular Media*, *11*, 227–236. <https://doi.org/10.1037/ppm0000352>

Arzy, S., Mohr, C., Molnar-Szakacs, I., & Blanke, O. (2011). Schizotypal perceptual aberrations of time: Correlation between score, behavior and brain activity. *PLoS ONE*, *6*, e16154. <https://doi.org/10.1371/journal.pone.0016154>

Ball, L., & Chandler, M. J. (1989). Identity formation in suicidal and nonsuicidal youth: The role of self-continuity. *Development and Psychopathology*, *1*, 257–275. <https://doi.org/10.1017/S0954579400000444>

Berenskötter, F. (2020). Anxiety, time, and agency. *International Theory*, *12*, 273–290. <https://doi.org/10.1017/S1752971920000111>

Biskas, M., Juhl, J., Wildschut, T., Sedikides, C., & Saroglou, V. (2022). Nostalgia and spirituality: The roles of self-continuity and meaning in life. *Social Psychology*, *53*, 152–162. <https://doi.org/10.1027/1864-9335/a000487>

Bizzari, V. (2023). Which kind of body in “mental” pathologies? Phenomenological insights on the nature of the disrupted self. *Journal of Medicine and Philosophy*, *48*, 116–127. <https://doi.org/10.1093/jmp/jhad008>

Blouin-Hudon, E. M. C., & Pychyl, T. A. (2017). A mental imagery intervention to increase future self-continuity and reduce procrastination. *Applied Psychology*, *66*, 326–352. <https://doi.org/10.1111/apps.12088>

Blouin-Hudon, E. M. C., Sirois, F. M., & Pychyl, T. A. (2016). Temporal views of procrastination, health, and well-being. In *Procrastination, health, and well-being* (pp. 213–232). Elsevier Inc. <https://doi.org/10.1016/B978-0-12-802862-9.00010-4>

Bluck, S., & Alea, N. (2009). Thinking and talking about the past: Why remember? *Applied Cognitive Psychology*, *23*, 1089–1104. <https://doi.org/10.1002/acp.1612>

Bluck, S., & Alea, N. (2011). Crafting the tale: Construction of a measure to assess the functions of autobiographical remembering. *Memory*, *19*, 470–486. <https://doi.org/10.1080/09658211.2011.590500>

Borda, J. P. (2016). Self over time: another difference between borderline personality disorder and bipolar disorder. *Journal of Evaluation in Clinical Practice*, *22*, 603–607. <https://doi.org/10.1111/jep.12550>

Bourke, K., de Abreu, G., & Rathbone, C. J. (2019). “I’m just who I am”: Self-continuity and the dialogical self in a study of migrants. *Journal of Constructivist Psychology*, *32*, 274–291. <https://doi.org/10.1080/10720537.2018.1488227>

Brinkers, M., Pfau, G., Thielmann, B., & Böckelmann, I. (2024). Cybernetic principles in psychophysiology: Their significance and conclusions for palliative care. *Healthcare*, *12*, 1510. <https://doi.org/10.3390/healthcare12151510>

Caci, B., Cardaci, M., & Miceli, S. (2019). Autobiographical memory, personality, and Facebook mementos. *Europe’s Journal of Psychology*, *15*, 614–636. <https://doi.org/10.5964/ejop.v15i3.1713>

Caci, B., Scrima, F., Cardaci, M., & Miceli, S. (2020). The Italian version of the Thinking About Life Experiences Questionnaire and its relationship with gender, age, and life events on Facebook. *Applied Cognitive Psychology*, *34*, 472–488. <https://doi.org/10.1002/acp.3635>

Callizo-Romero, C., Tutnjević, S., Pandza, M., Ouellet, M., Kranjec, A., Ilić, S., Gu, Y., Göksun, T., Chahboun, S., Casasanto, D., & Santiago, J. (2022). Does time extend asymmetrically into the past and the future? A multitask crosscultural study. *Language and Cognition*, *14*, 275–302. <https://doi.org/10.1017/langcog.2022.5>

Camia, C., & Zafar, R. (2021). Autobiographical meaning making protects the sense of self-continuity past forced migration. *Frontiers in Psychology*, *12,* 618343. <https://doi.org/10.3389/fpsyg.2021.618343>

Chandler, M. J. (1994). Self‐continuity in suicidal and nonsuicidal adolescents. *New Directions for Child and Adolescent Development*, *1994*, 55–70. <https://doi.org/10.1002/cd.23219946406>

Chandler, M. J. (2000). Surviving time: The persistence of identity in this culture and that. *Culture & Psychology*, *6*, 209–231. <https://doi.org/10.1177/1354067X0062009>

Chandler, M. J. (2001). The time of our lives: Self-continuity in Native and non-Native youth. In H. W. Reese & R. Kail (Eds.), *Advances in child development and behavior* (pp. 175–221). Academic Press. <https://doi.org/10.1016/S0065-2407(02)80065-9>

Chandler, M. J., Boyes, M., Ball, L., & Hala, S. (1987). The conservation of selfhood: A developmental analysis of children’s changing conceptions of self-continuity. In T. Honess & K. Yardley (Eds.), *Self and identity: Perspectives across the lifespan* (pp. 108–133). Routledge.

Chandler, M. J., & Lalonde, C. E. (1995). The problem of self-continuity in the context of rapid personal and cultural change. In A. Oosterwegel & R. A. Wicklund (Eds.), *Proceedings of the NATO advanced research workshop on the self in European and North American culture: Development and processes* (pp. 45–64). Springer-Science+Business Media. <https://doi.org/10.1007/978-94-011-0331-2>

Chandler, M. J., & Lalonde, C. E. (1998). Cultural continuity as a hedge against suicide in Canada’s first nations. *Transcultural Psychiatry*, *35*, 191–219. <https://doi.org/10.1177/136346159803500202>

Chandler, M. J., Lalonde, C. E., & Sokol, B. (2000). Continuities of selfhood in the face of radical development and cultural change. In L. Nucci, G. Saxe, & E. Turiel (Eds.), *Culture, thought and development* (pp. 1–25). Psychology Press. <https://doi.org/10.4324/9781410603470>

Chandler, M. J., Lalonde, C. E., Sokol, B. W., Hallett, D., & Marcia, J. E. (2003). Personal persistence, identity development, and suicide: A study of Native and Non-Native North American Adolescents. *Monographs of the Society for Research in Child Development*, *68*, 1–138. <https://doi.org/10.1111/1540-5834.00246>

Chandler, M. J., & Proulx, T. (2008). Personal persistence and persistent peoples: Continuities in the lives of individual and whole cultural communities. In F. Sani (Ed.), *Self continuity: Individual and collective perspectives* (pp. 213–226). Psychology Press.

Chishima, Y., & Wilson, A. E. (2021). Conversation with a future self: A letter-exchange exercise enhances student self-continuity, career planning, and academic thinking. *Self and Identity, 20*, 646–671. [https://doi.org/10.1080/15298868.2020.1754283](https://psycnet.apa.org/doi/10.1080/15298868.2020.1754283)

Cho, H., Li, P., & Chen, A. (2024). Nostalgia, social media, and subjective wellbeing: The dualistic and conditional effects of nostalgia during the COVID-19 pandemic. *Health Communication*, *39*, 507–517. <https://doi.org/10.1080/10410236.2023.2170723>

Clare, L., Martyr, A., Morris, R. G., & Tippett, L. J. (2020). Discontinuity in the subjective experience of self among people with mild-to-moderate dementia is associated with poorer psychological health: Findings from the IDEAL cohort. *Journal of Alzheimer’s Disease*, *77*, 127–138. <https://doi.org/10.3233/JAD-200407>

Cory, T. S. (2012). Diachronically unified consciousness in Augustine and Aquinas. *Vivarium*, *50*, 354–381. <https://doi.org/10.1163/15685349-12341237>

Craig, A. D. B. (2009a). Emotional moments across time: A possible neural basis for time perception in the anterior insula. *Philosophical Transactions of the Royal Society B: Biological Sciences*, *364*, 1933–1942. <https://doi.org/10.1098/rstb.2009.0008>

Craig, A. D. B. (2009b). How do you feel — now? The anterior insula and human awareness. *Nature Reviews Neuroscience*, *10,* 59–70. <https://doi.org/10.1038/nrn2555>

Dai, Y., Li, Q., Zhou, H., & Jiang, T. (2023). Nostalgia and online autobiography: Implications for global self-continuity and psychological well-being. *Journal of Happiness Studies: An Interdisciplinary Forum on Subjective Well-Being, 24*, 2747–2763. [https://doi.org/10.1007/s10902-023-00701-y](https://psycnet.apa.org/doi/10.1007/s10902-023-00701-y)

Dary, Z., & Lopez, C. (2023). Understanding the neural bases of bodily self-consciousness: Recent achievements and main challenges. *Frontiers in Integrative Neuroscience*, *17*, 1145924. <https://doi.org/10.3389/fnint.2023.1145924>

Davis, O. M. (2020). *Who am I: Sense of self in individuals with sudden-onset episodic amnesia* [Unpublished master’s thesis]. University of Auckland.

de la Mata-Benítez, M. L., Español, A., Matías-García, J. A., Lojo, M., & Villar-Toribio, C. del. (2024). Repairing the breach: Identity narratives of a Latin American woman in Andalusia. *Culture and Psychology*, *30*, 150–172. <https://doi.org/10.1177/1354067X231160237>

Dorahy, M. J., Huntjens, R. J. C., Marsh, R. J., Johnson, B., Fox, K., & Middleton, W. (2021). The sense of self over time: Assessing diachronicity in dissociative identity disorder, psychosis and healthy comparison groups. *Frontiers in Psychology*, *12*, 620063. <https://doi.org/10.3389/fpsyg.2021.620063>

Dowd, A. (2020). Displacement trauma: Complex states of personal, collective and intergenerational fragmentation and their intergenerational transmission. *Journal of Analytical Psychology*, *65*, 300–324. <https://doi.org/10.1111/1468-5922.12588>

Droit-Volet, S., & Dambrun, M. (2019). Awareness of the passage of time and self-consciousness: What do meditators report? *PsyCh Journal*, *8*, 51–65. <https://doi.org/10.1002/pchj.270>

Dunlop, W. L., & Walker, L. J. (2015). Cross-cultural variability in self-continuity warranting strategies. *Journal of Language and Social Psychology*, *34*, 300–315. <https://doi.org/10.1177/0261927X14555873>

El Haj, M., & Allain, P. (2020). Self-defining memories and their contribution to the sense of self in Alzheimer’s disease. *Current Alzheimer Research*, *17*, 508–516. <https://doi.org/10.2174/1567205017666200807184942>

El Haj, M., Boudoukha, A., Antoine, P., Moustafa, A. A., Gallouj, K., & Allain, P. (2019). Memories supporting myself: Autobiographical memory supports self-continuity in Alzheimer’s disease. *Journal of Alzheimer’s Disease*, *70*, 1217–1224. <https://doi.org/10.3233/JAD-190440>

Eli, K. (2016). The body remembers: Narrating embodied reconciliations of eating disorder and recovery. *Anthropology and Medicine*, *23*, 71–85. <https://doi.org/10.1080/13648470.2015.1135786>

Elkins, J. (2015). Motility, aggression, and the bodily I: An interpretation of Winnicott. *Psychoanalytic Quarterly*, *84*, 943–973. <https://doi.org/10.1002/psaq.12044>

Evans, N. D. (2022). *Language dependent effects of nostalgia, via mental transportation, among bilinguals* [Unpublished PhD thesis]. The University of Texas at El Paso.

Fingelkurts, A. A., Fingelkurts, A. A., & Kallio-Tamminen, T. (2016). Long-term meditation training induced changes in the operational synchrony of default mode network modules during a resting state. *Cognitive Processing*, *17*, 27–37. <https://doi.org/10.1007/s10339-015-0743-4>

Fuchs, T. (2013). Temporality and psychopathology. *Phenomenology and the Cognitive Sciences*, *12*, 75–104. <https://doi.org/10.1007/s11097-010-9189-4>

Fuchs, T. (2017). Self across time: The diachronic unity of bodily existence. *Phenomenology and the Cognitive Sciences*, *16*, 291–315. <https://doi.org/10.1007/s11097-015-9449-4>

Fuchs, T. (2018). The cyclical time of the body and its relation to linear time. *Journal of Consciousness Studies*, *25*, 47–65.

Ganschow, B., Cornet, L., Zebel, S., & van Gelder, J.-L. (2021). Looking back from the future: perspective taking in virtual reality increases future self-continuity. *Frontiers in Psychology*, *12,* 664687. <https://doi.org/10.3389/fpsyg.2021.664687>

Godoy-Casasbuenas, N., Cadena-Camargo, Y., Rodríguez, N., de Vries, E., & Yaron, G. (2024). “You can be cured, but cancer never leaves you behind”: An interdisciplinary approach into the embodied cancer experiences among adult Colombian childhood/adolescent cancer survivors. *Journal of Psychosocial Oncology*, *42*, 859–874. <https://doi.org/10.1080/07347332.2024.2345112>

Grabowski, A., & Broemer, P. (2015). Temporal self-extension: Implications for temporal comparison and autobiographical memory. *Polish Psychological Bulletin*, *46*, 246–261. <https://doi.org/10.1515/ppb-2015-0033>

Grandits, C. (2020). *Diachronic unity and basic needs satisfaction: Linking self-determination theory with continuous identity* [Unpublished PhD thesis]. Hofstra University.

Habermas, T., & Köber, C. (2015a). Autobiographical reasoning in life narratives buffers the effect of biographical disruptions on the sense of self-continuity. *Memory*, *23*, 664–674. <https://doi.org/10.1080/09658211.2014.920885>

Habermas, T., & Köber, C. (2015b). Autobiographical reasoning is constitutive for narrative identity: The role of the life story for personal continuity. In K. C. McLean & M. Syed (Eds.), *The Oxford handbook of identity development* (pp. 149–165). Oxford University Press.

Harris, S. A., Baird, A., Matthews, S., Kennett, J., Gelding, R., & Harris, C. B. (2021). The impact of dementia on the self: Do we consider ourselves the same as others? *Neuroethics, 14*, 281–294. [https://doi.org/10.1007/s12152-021-09472-w](https://psycnet.apa.org/doi/10.1007/s12152-021-09472-w)

Hazan, H., Reese, E. J., & Linscott, R. J. (2019). Narrative self and high risk for schizophrenia: remembering the past and imagining the future. *Memory*, *27*, 1214–1223. <https://doi.org/10.1080/09658211.2019.1642919>

Hill, J. (1996). At home in the world. *Journal of Analytical Psychology*, *41*, 575–598. <https://doi.org/10.1111/j.1465-5922.1996.00575.x>

Hong, E. K., Sedikides, C., & Wildschut, T. (2021). Nostalgia strengthens global self-continuity through holistic thinking. *Cognition and Emotion*, *35*, 730–737. <https://doi.org/10.1080/02699931.2020.1862064>

Hong, E. K., Sedikides, C., & Wildschut, T. (2022). How does nostalgia conduce to global self-continuity? The roles of identity narrative, associative links, and stability. *Personality and Social Psychology Bulletin*, *48*, 735–749. <https://doi.org/10.1177/01461672211024889>

Izadifar, M. (2022). The neurobiological basis of the conundrum of self-continuity: A hypothesis. *Frontiers in Psychology*, *13*, 740542. <https://doi.org/10.3389/fpsyg.2022.740542>

Jacard, S. M. (2001). *The impact of immigration on an individual’s sense of self* [Unpublished PhD thesis]. Massachusetts School of Professional Psychology.

Ji, L. J., Hong, E. K., Guo, T., Zhang, Z., Su, Y., & Li, Y. (2018). Culture, psychological proximity to the past and future, and self-continuity. *European Journal of Social Psychology*, *49*, 735–747. <https://doi.org/10.1002/ejsp.2544>

Ji, L. J., Imtiaz, F., Su, Y., Zhang, Z., Bowie, A. C., & Chang, B. (2022). Culture, aging, self-continuity, and life satisfaction. *Journal of Happiness Studies*, *23*, 3843–3864. <https://doi.org/10.1007/s10902-022-00568-5>

Jiang, T., Chen, Z., & Sedikides, C. (2020). Self-concept clarity lays the foundation for self-continuity: The restorative function of autobiographical memory. *Journal of Personality and Social Psychology*, *119*, 945–959. <https://doi.org/10.1037/pspp0000259>

Jiang, T., Chen, Z., Wang, S., & Hou, Y. (2021). Ostracism disrupts self-continuity. *Personality and Social Psychology Bulletin*, *47*, 1390–1400. <https://doi.org/10.1177/0146167220974496>

Jobson, L., Whittles, N., Tsecoutanis, E., Raj, S., Yew, R. Y., & Haque, S. (2019). Investigating the mediating role of self-construal on the relationship between cultural group (Malay and Australian) and the characteristics and functional use of autobiographical memory. *Memory*, *27*, 1054–1062. <https://doi.org/10.1080/09658211.2019.1619776>

Juhl, J., & Biskas, M. (2023). Nostalgia: An impactful social emotion. *Current Opinion in Psychology*, *49*, 101545. <https://doi.org/10.1016/j.copsyc.2022.101545>

Klein, S. B. (2014). Sameness and the self: Philosophical and psychological considerations. *Frontiers in Psychology*, *5*, 29. <https://doi.org/10.3389/fpsyg.2014.00029>

Lalonde, C. E., & Chandler, M. J. (2004). Culture, selves, and time: Theories of personal persistence in native and non-native youth. In C. Lightfoot, M. J. Chandler, & C. E. Lalonde (Eds.), *Changing conceptions of psychological life* (pp. 207–229). Psychology Press. <https://doi.org/10.4324/9781410610966-16>

Lampraki, C., Spini, D., & Jopp, D. S. (2023). Life span development of self-continuity in late adulthood: The impact of chronological age, loss of partner and aversive childhood events. *Self and Identity*, *22*, 669–688. <https://doi.org/10.1080/15298868.2022.2157869>

Lasaleta, J. D., & Loveland, K. E. (2019). What’s new is old again: Nostalgia and retro-styling in response to authenticity threats. *Journal of the Association for Consumer Research*, *4*, 172–184. <https://doi.org/10.1086/702473>

Layous, K., Kurtz, J. L., Wildschut, T., & Sedikides, C. (2021). The effect of a multi-week nostalgia intervention on well-being: Mechanisms and moderation. *Emotion*, *22*, 1952–1968. <https://doi.org/10.1037/emo0000817>

Lee, A. R. (2017). Psychological proximity to issues of the elderly: The role of age-morphing technology in campaigns for the elderly. *Computers in Human Behavior*, *69*, 311–323. <https://doi.org/10.1016/j.chb.2016.12.046>

Lee-Won, R. J., Lee, E. J., & Lee, J. A. (2023). Nostalgic social media use and psychological well-being during the COVID-19 pandemic. *Cyberpsychology, Behavior, and Social Networking*, *26*, 90–97. <https://doi.org/10.1089/cyber.2022.0281>

Levin, C., Nenninger, S., Freundlich, D., Glatt, S., & Sokol, Y. (2023). How future self-continuity mediates the impact of job loss on negative mental health outcomes among transitioning veterans. *Military Psychology*, *36*, 491–503. <https://doi.org/10.1080/08995605.2023.2215697>

Lewin, R. A. (1991). Preliminary thoughts on milieu treatment of patients with multiple personality disorder. *The Psychiatric Hospital*, *22*, 161–163.

Leyva, A. (2022). Embodied movement consciousness. *Phenomenology and the Cognitive Sciences*, *21*, 161–180. <https://doi.org/10.1007/s11097-021-09792-5>

Liao, H.-W. (2017). *Being me over time: The self-continuity function of autobiographical memory in adulthood* [PhD thesis, University of Florida]. ProQuest Dissertations & Theses Global.

Liao, H.-W., & Bluck, S. (2022). Recalling self-disruptive events and maintaining self-continuity in adulthood. *Psychology and Aging*, *38*, 17–29. <https://doi.org/10.1037/pag0000719>

Liao, H.-W., Bluck, S., Alea, N., & Cheng, C. L. (2016). Functions of autobiographical memory in Taiwanese and American emerging adults. *Memory*, *24*, 423–436. <https://doi.org/10.1080/09658211.2015.1015572>

Lin, Y. S., Hartwich, P., Wolff, A., Golesorkhi, M., & Northoff, G. (2020). The self in art therapy – Brain-based assessment of the drawing process. *Medical Hypotheses*, *138*, 109596. <https://doi.org/10.1016/j.mehy.2020.109596>

Lind, M., Bluck, S., & Åkerlund, H. (2020). Adults’ memories of childhood: The beginning of the life story. In S. Gulgoz & B. Sahin-Acar (Eds.), *Autobiographical memory development: Theoretical and methodological approaches* (1st ed., pp. 148–160). Routledge. <https://doi.org/10.4324/9780429022791-12>

Löckenhoff, C. E., & Rutt, J. L. (2017). Age differences in self-continuity: Converging evidence and directions for future research. *Gerontologist*, *57*, 396–408. <https://doi.org/10.1093/geront/gnx010>

Low, K. E. Y. (2013). Olfactive frames of remembering: Theorizing self, senses and society. *Sociological Review*, *61*, 688–708. <https://doi.org/10.1111/1467-954X.12078>

Lu, Y., Gerstorf, D., & Löckenhoff, C. E. (2023). Age differences in self-continuity in Germany and the United States: The role of temporal direction, temporal distance, and demographics. *Journals of Gerontology - Series B Psychological Sciences and Social Sciences*, *78*, 841–846. <https://doi.org/10.1093/geronb/gbad002>

Martin, B., Wittmann, M., Franck, N., Cermolacce, M., Berna, F., & Giersch, A. (2014). Temporal structure of consciousness and minimal self in schizophrenia. *Frontiers in Psychology*, *5*, 1175. https://doi.org/10.3389/fpsyg.2014.01175

Martin-Storey, A., Recchia, H. E., & Santo, J. B. (2021). Self-continuity moderates the association between sexual-minority status based discrimination and depressive symptoms. *Journal of Homosexuality*, *68*, 2075–2096. <https://doi.org/10.1080/00918369.2020.1733350>

Märtsin, M., & Mahmoud, H. W. (2012). Never at home? Migrants between societies. In J. Valsiner (Ed.), *The Oxford handbook of culture and psychology* (pp. 730–745). Oxford University Press. <https://doi.org/10.1093/oxfordhb/9780195396430.013.0035>

Mellor, A., Surrounded by Cedar Child and Family Services, & Cloutier, D. (2020). Becoming self-in-relation: Coming of age as a pathway towards wellness for urban Indigenous youth in care. *First Peoples Child & Family Review*, *15*, 1–22. <https://doi.org/10.7202/1080807ar>

Mensch, J. (2015). Desire and selfhood. *European Legacy*, *20*, 689–698. <https://doi.org/10.1080/10848770.2015.1067961>

Mertens, E. C. A., Siezenga, A. M., Tettero, T., & van Gelder, J. L. (2022). A future orientation intervention delivered through a smartphone application and virtual reality: study protocol for a randomized controlled trial. *BMC Psychology*, *10*, 315. <https://doi.org/10.1186/s40359-022-01025-x>

Mertens, E. C. A., van der Schalk, J., Siezenga, A. M., & van Gelder, J. L. (2022). Stimulating a future-oriented mindset and goal attainment through a smartphone-based intervention: Study protocol for a randomized controlled trial. *Internet Interventions*, *27,* 100509. <https://doi.org/10.1016/j.invent.2022.100509>

Monti, A., Porciello, G., Panasiti, M. S., & Aglioti, S. M. (2022). The inside of me: Interoceptive constraints on the concept of self in neuroscience and clinical psychology. *Psychological Research*, *86*, 2468–2477. <https://doi.org/10.1007/s00426-021-01477-7>

Naisani Samani, M., & Busseri, M. A. (2019). Examining the link between mindfulness and temporal perspective. *Mindfulness*, *10*, 2647–2660. <https://doi.org/10.1007/s12671-019-01240-5>

Northoff, G. (2020). Habits and self: A temporal view. In F. Caruana & I. Testa (Eds.), *Habits: Pragmatist approaches from cognitive science, neuroscience, and social theory* (pp. 58–78). Cambridge University Press. <https://doi.org/10.1017/9781108682312.003>

Nyholm, S., & O’Neill, E. (2016). Deep brain stimulation, continuity over time, and the true self. *Cambridge Quarterly of Healthcare Ethics*, *25*, 647–658. <https://doi.org/10.1017/S0963180116000372>

Owen, S., Page, S., Ledingham, K., Price, S., Connell, J., Quinn, C., & Clare, L. (2024). Embodied leisure experiences of nature-based activities for people living with dementia. *Dementia*, *23*, 1081–1102. <https://doi.org/10.1177/14713012241262384>

Pachana, N. A., & Baumeister, R. F. (2021). Better later than never: Meaning in late life. *Frontiers in Psychology*, *12*, 693116. <https://doi.org/10.3389/fpsyg.2021.693116>

Pan, X., & Jiang, T. (2022). Awe fosters global self-continuity: The mediating effect of global processing and narrative. *Emotion*, *23*, 1618–1632. <https://doi.org/10.1037/emo0001187>

Park, H. D., & Blanke, O. (2019). Coupling inner and outer body for self-consciousness. *Trends in Cognitive Sciences*, *23*, 377–388. <https://doi.org/10.1016/j.tics.2019.02.002>

Park, H. D., & Tallon-Baudry, C. (2014). The neural subjective frame: From bodily signals to perceptual consciousness. *Philosophical Transactions of the Royal Society B: Biological Sciences*, *369*, 1–9. <https://doi.org/10.1098/rstb.2013.0208>

Parvizi-Wayne, D., Sandved-Smith, L., Pitliya, R. J., Limanowski, J., Tufft, M. R. A., & Friston, K. J. (2024). Forgetting ourselves in flow: an active inference account of flow states and how we experience ourselves within them. *Frontiers in Psychology*, *15*, 1354719. <https://doi.org/10.3389/fpsyg.2024.1354719>

Prebble, S. C., Addis, D. R., & Tippett, L. J. (2013). Autobiographical memory and sense of self. *Psychological Bulletin*, *139*, 815–840. <https://doi.org/10.1037/a0030146>

Putnam, F. W. (1994). Dissociation and disturbances of self. In D. Cicchetti & S. L. Toth (Eds.), *Rochester symposium on developmental psychopathology: Disorders and dysfunctions of the self* (Vol. 5, pp. 251–265). University of Rochester Press.

Raby, C. R., & Clayton, N. S. (2012). Episodic memory and planning. In T. K. Shackelford & J. Vonk (Eds.), *The Oxford handbook of comparative evolutionary psychology* (pp. 217–235). Oxford University Press. <https://doi.org/10.1093/oxfordhb/9780199738182.013.0012>

Rashed, M. A. (2021). An approach to the boundary problem: Mental health activism and the limits of recognition. *Philosophy, Psychiatry and Psychology*, *28*, 297–313. <https://doi.org/10.1353/ppp.2021.0047>

Ratner, K., & Burrow, A. L. (2021). Derailment within the landscape of psychopathology. *Current Opinion in Psychology*, *41*, 21–27. <https://doi.org/10.1016/j.copsyc.2021.01.009>

Revach, D., & Salti, M. (2022). Consciousness as the temporal propagation of information. *Frontiers in Systems Neuroscience*, *16*, 759683. <https://doi.org/10.3389/fnsys.2022.759683>

Ritchie, T. D., Sedikides, C., Wildschut, T., Arndt, J., & Gidron, Y. (2011). Self-concept clarity mediates the relation between stress and subjective well-being. *Self and Identity*, *10*, 493–508. <https://doi.org/10.1080/15298868.2010.493066>

Rutt, J. L., & Löckenhoff, C. E. (2016). Age patterns in mental representations of time: Underlying constructs and relevant covariates. *Experimental Aging Research*, *42*, 289–306. <https://doi.org/10.1080/0361073X.2016.1156975>

Salgado, J., Cunha, C., & Bento, T. (2013). Positioning microanalysis: Studying the self through the exploration of dialogical processes. *Integrative Psychological and Behavioral Science*, *47*, 325–353. <https://doi.org/10.1007/s12124-013-9238-y>

Sani, F. (2008). Introduction and overview. In F. Sani (Ed.), *Self-continuity: Individual and collective perspectives* (pp. 1-12). Psychology Press.

Santo, J. B., da Cunha, J., Saldarriaga, L. M., Recchia, H. E., Martin-Storey, A., Stella-Lopez, L., Carmago, G., & Bukowski, W. M. (2013). Measurement invariance of self-continuity strategies: Comparisons of early adolescents from Brazil, Canada and Colombia. *European Journal of Developmental Psychology*, *10*, 518–525. <https://doi.org/10.1080/17405629.2012.707313>

Scalabrini, A., Wolman, A., & Northoff, G. (2021). The self and its right insula—Differential topography and dynamic of right vs. left insula. *Brain Sciences*, *11*, 1312. <https://doi.org/10.3389/fnhum.2022.980353>

Sedikides, C., Hong, E. K., & Wildschut, T. (2023). Self-continuity. *Annual Reviews of Psychology*, *74*, 333–361. <https://doi.org/10.1146/annurev-psych-032420>

Sedikides, C., & Wildschut, T. (2018). Finding meaning in Nostalgia. *Review of General Psychology*, *22*, 48–61. <https://doi.org/10.1037/gpr0000109>

Sedikides, C., & Wildschut, T. (2019). The sociality of personal and collective nostalgia. *European Review of Social Psychology*, *30*, 123–173. <https://doi.org/10.1080/10463283.2019.1630098>

Sedikides, C., & Wildschut, T. (2022). Nostalgia across cultures. *Journal of Pacific Rim Psychology*, *16*, 1–16. <https://doi.org/10.1177/18344909221091649>

Sedikides, C., Wildschut, T., Cheung, W. Y., Hepper, E. G., Vail, K., Brackstone, K., Routledge, C., Arndt, J., Zhou, X., & Vingerhoets, A. J. J. M. (2016). Nostalgia fosters self-continuity: Uncovering the mechanism (social connectedness) and consequence (eudaimonic well-being). *Emotion*, *16*, 524–539. <https://doi.org/10.1037/emo0000136>

Sedikides, C., Wildschut, T., & Grouzet, F. (2018). On the temporal navigation of selfhood: The role of self-continuity. *Self and Identity*, *17*, 255–258. <https://doi.org/10.1080/15298868.2017.1391115>

Sedikides, C., Wildschut, T., Routledge, C., & Arndt, J. (2015). Nostalgia counteracts self-discontinuity and restores self-continuity. *European Journal of Social Psychology*, *45*, 52–61. <https://doi.org/10.1002/ejsp.2073>

Seth, A. K., & Tsakiris, M. (2018). Being a beast machine: The somatic basis of selfhood. *Trends in Cognitive Sciences*, *22*, 969–981. <https://doi.org/10.1016/j.tics.2018.08.008>

Shen, Y. I., Nelson, A. J., & Oberlin, B. G. (2022). Virtual reality intervention effects on future self-continuity and delayed reward preference in substance use disorder recovery: pilot study results. *Discover Mental Health*, *2*, 19. <https://doi.org/10.1007/s44192-022-00022-1>

Skirrow, P., Jackson, P., Perry, E., & Hare, D. J. (2015). I collect therefore I am-Autonoetic consciousness and hoarding in Asperger syndrome. *Clinical Psychology and Psychotherapy*, *22*, 278–284. <https://doi.org/10.1002/cpp.1889>

Smith, A. H. (1987). Much ado about nothing: On the integration of non-being into the self in obsessed states. *Psychotherapy and the Obsessed Patient*, *3*, 85–99.

Sokol, Y., Conroy, A. K., & Weingartner, K. M. (2017). The cognitive underpinnings of continuous identity: Higher episodic memory recall and lower heuristic usage predicts highest levels of self-continuity. *Identity*, *17*, 84–95. <https://doi.org/10.1080/15283488.2017.1303384>

Sokol, Y., & Serper, M. R. (2019). Temporal self, psychopathology, and adaptive functioning deficits: An examination of acute psychiatric patients. *Journal of Nervous and Mental Disease*, *207*, 76–83. <https://doi.org/10.1097/NMD.0000000000000925>

Stern, Y., Ben-Yehuda, I., Koren, D., Zaidel, A., & Salomon, R. (2022). The dynamic boundaries of the self: Serial dependence in the sense of agency. *Cortex*, *152*, 109–121. <https://doi.org/10.1016/j.cortex.2022.03.015>

Syed, M., & Fish, J. (2018). Revisiting Erik Erikson’s legacy on culture, race, and ethnicity. *Identity*, *18*, 274–283. <https://doi.org/10.1080/15283488.2018.1523729>

Tafarodi, R. W., Lo, C., Yamaguchi, S., Lee, W. W. S., & Katsura, H. (2004). The inner self in three countries. *Journal of Cross-Cultural Psychology*, *35*, 97–117. <https://doi.org/10.1177/0022022103260462>

Tanguay, A. N., Palombo, D. J., Atance, C. M., Renoult, L., & Davidson, P. S. R. (2020). Scrutinizing the grey areas of declarative memory: Do the self-reference and temporal orientation of a trait knowledge task modulate the Late Positive Component (LPC)? *Neuropsychologia*, *142*, 107444. <https://doi.org/10.1016/j.neuropsychologia.2020.107444>

Tausen, B. M., Csordas, A., & MacRae, C. N. (2020). The mental landscape of imagining life beyond the current life span: Implications for construal and self-continuity. *Innovation in Aging*, *4*, 1–16. <https://doi.org/10.1093/geroni/igaa013>

Taylor, J. G. (2007). On the neurodynamics of the creation of consciousness. *Cognitive Neurodynamics*, *1*, 97–118. <https://doi.org/10.1007/s11571-006-9011-8>

Tippett, L. J., Prebble, S. C., & Addis, D. R. (2018). The persistence of the self over time in mild cognitive impairment and Alzheimer’s disease. *Frontiers in Psychology*, *9,* 94. <https://doi.org/10.3389/fpsyg.2018.00094>

Tisserand, A., Philippi, N., Botzung, A., & Blanc, F. (2023). Me, myself and my insula: An oasis in the forefront of self-consciousness. *Biology*, *12*, 599. <https://doi.org/10.3390/biology12040599>

Tomán, E., Pintér, J. N., & Hargitai, R. (2024). The role of the lived body during the integration of the traumatic experience of the sternotomy scar: A case study. *Journal of Evaluation in Clinical Practice*, *30*, 804–816. <https://doi.org/10.1111/jep.13925>

Troll, L. E., & Skaff, M. M. (1997). Perceived continuity of self in very old age. *Psychology and Aging*, *12*, 162–169. <https://doi.org/10.1037/0882-7974.12.1.162>

Uriko, K., & Bartels, I. (2023). Separation in unity: Dialogical transformation of maternal bond. *Integrative Psychological and Behavioral Science*, *57*, 590–606. <https://doi.org/10.1007/s12124-022-09688-2>

van Tilburg, W. A. P., Sedikides, C., Wildschut, T., & Vingerhoets, A. J. J. M. (2019). How nostalgia infuses life with meaning: From social connectedness to self-continuity. *European Journal of Social Psychology*, *49*, 521–532. <https://doi.org/10.1002/ejsp.2519>

Walker, S. (2017). *Stroke, self, identity and post-stroke psychological distress* [Unpublished PhD thesis]. University of Auckland.

Ward, H. (2011). Continuities and discontinuities: Issues concerning the establishment of a persistent sense of self amongst care leavers. *Children and Youth Services Review*, *33*, 2512–2518. <https://doi.org/10.1016/j.childyouth.2011.08.028>

Waskul, D. D., Vannini, P., & Wilson, J. (2009). The aroma of recollection: Olfaction, nostalgia, and the shaping of the sensuous self. *Senses and Society*, *4*, 5–22. <https://doi.org/10.2752/174589309X388546>

Weiler, M., Northoff, G., Damasceno, B. P., & Balthazar, M. L. F. (2016). Self, cortical midline structures and the resting state: Implications for Alzheimer’s disease. *Neuroscience and Biobehavioral Reviews*, *68*, 245–255. <https://doi.org/10.1016/j.neubiorev.2016.05.028>

Wildschut, T., Sedikides, C., & Robertson, S. (2018). Sociality and intergenerational transfer of older adults’ nostalgia. *Memory*, *26*, 1030–1041. <https://doi.org/10.1080/09658211.2018.1470645>

Wilson, S. G. (2014). Enhancement and identity: A social psychological perspective. In S. J. Thompson (Ed.), *Global issues and ethical considerations in human enhancement technologies* (pp. 241–256). IGI Global. <https://doi.org/10.4018/978-1-4666-6010-6.ch014>

Wolf, T., & Zimprich, D. (2016). The distribution and the functions of autobiographical memories: Why do older adults remember autobiographical memories from their youth? *European Journal of Ageing*, *13*, 241–250. <https://doi.org/10.1007/s10433-016-0372-5>

Zelazo, P. D., & Sommerville, J. A. (2001). Levels of consciousness of the self in time. In C. Moore & K. Lemmon (Eds.), *The self in time: Developmental perspectives* (pp. 229–252). Taylor & Francis Group.

Zittoun, T., Grossen, M., & Tarrago Salamin, F. (2021). Creating new spheres of experience in the transition to a nursing home. *Learning, Culture and Social Interaction*, *28*, 100458. <https://doi.org/10.1016/j.lcsi.2020.100458>

Zou, X., & Petkanopoulou, K. (2023). Nostalgia and acculturation. *Current Opinion in Psychology*, *49*, 101553. https://doi.org/10.1016/j.copsyc.2022.101553
